# Competence induction of homologous recombination genes protects pneumococcal cells from genotoxic stress

**DOI:** 10.1128/mbio.03142-24

**Published:** 2024-11-29

**Authors:** David De Lemos, Anne-Lise Soulet, Violette Morales, Mathieu Berge, Patrice Polard, Calum Johnston

**Affiliations:** 1 Laboratoire de Microbiologie et Génétique Moléculaires (LMGM), UMR5100, Centre de Biologie Intégrative (CBI), Centre Nationale de la Recherche Scientifique (CNRS), Toulouse, France; 2 Université Paul Sabatier (Toulouse III), Toulouse, France; Carnegie Mellon University, Pittsburgh, Pennsylvania, USA

**Keywords:** homologous recombination, transformation, genome maintenance, competence, tolerance

## Abstract

Homologous recombination (HR) is a universally conserved mechanism of DNA strand exchange between homologous sequences, driven in bacteria by the RecA recombinase. HR is key for the maintenance of bacterial genomes via replication fork restart and DNA repair, as well as for their plasticity via the widespread mechanism of natural transformation. Transformation involves the capture and internalization of exogenous DNA in the form of single strands, followed by HR-mediated chromosomal integration. In the human pathogen *Streptococcus pneumoniae*, transformation occurs during a transient, stress-induced differentiation state called competence. RecA and its partner DNA branch migration translocase RadA are both well conserved and cooperate in HR during transformation and certain genome maintenance pathways. Both *recA* and *radA* genes are basally expressed and transcriptionally induced during competence. In this study, we explored the importance of competence induction of *recA* and *radA* in transformation and genome maintenance. We confirmed that competence induction of *recA*, but not radA, was important for transformation. In contrast, we uncovered that the competence induction of both genes was required for optimal tolerance faced with transient exposure to the lethal genotoxic agent methyl methanesulfonate. However, this was not the case for another DNA-damaging agent, norfloxacin. These results show that competence induction of HR effectors is important for the increased tolerance to genotoxic stress provided to competent pneumococci. This reinforces the finding that pneumococcal competence is a stress-sensing mechanism, transiently increasing the expression of some genes not to optimize transformation but to improve survival faced with specific lethal stresses.

**IMPORTANCE:**

Homologous recombination (HR) is a mechanism of DNA strand exchange important for both the maintenance and plasticity of bacterial genomes. Bacterial HR is driven by the recombinase RecA along with many accessory partner proteins, which define multiple dedicated pathways crucial to genome biology. Thus, a main mechanism of genome plasticity in bacteria is natural genetic transformation, which involves uptake and chromosomal integration of exogenous DNA via HR. In the human pathogen *Streptococcus pneumoniae*, transformation occurs during a transient, stress-induced physiological state called competence. RecA and the helicase RadA are key for both genome maintenance and transformation, and both are over-produced during competence. Here, we explore the importance of this over-production for transformation and genome maintenance, quantified by tolerance to genotoxic stress. While over-production of RecA was important for both processes, over-production of RadA was required only for genotoxic stress tolerance. This highlights the importance of competence as a stress-responsive mechanism, with induction of HR genes important for genotoxic stress tolerance.

## INTRODUCTION

**Fig 1:**
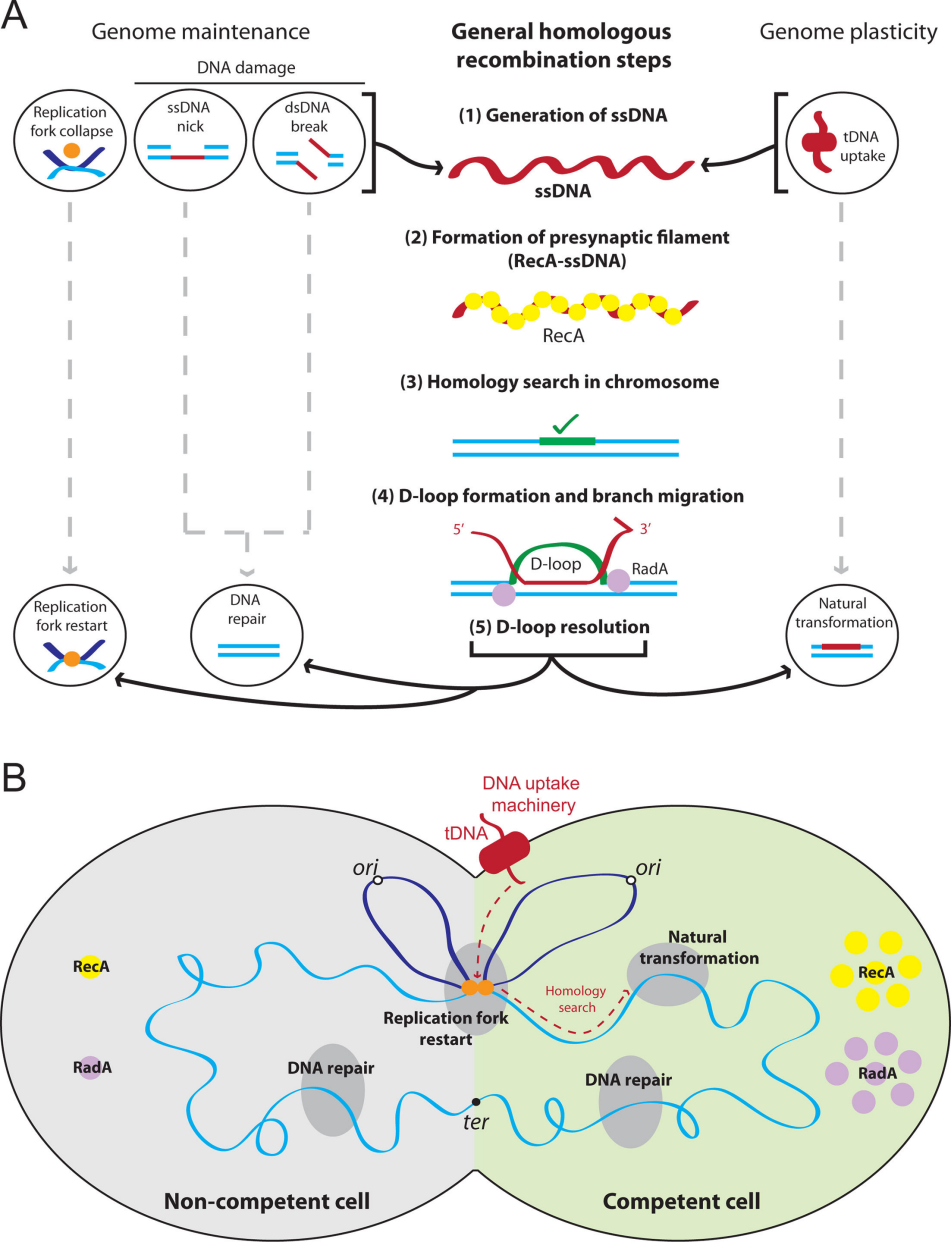
HR pathways in competent and non-competent pneumococcal cells. (**A**) General steps conserved among HR pathways in the pneumococcus. (1) The first step of HR involves the generation of ssDNA within the cell, which can occur via replication fork collapse, DNA damage (ssDNA nicks or dsDNA breaks), or uptake of ssDNA during the genome plasticity mechanism of natural transformation. (2) The homologous recombinase RecA is then loaded onto the ssDNA, and polymerizes to form a presynaptic filament. (3) RecA mediates homology search within the chromosome. (4) Strand exchange between homologous sequences forms a recombination intermediate known as a D-loop, with branch migration by helicases including RadA extending this structure. (5) Further processing and D-loop resolution results in replication fork restart, DNA repair, or integration of transforming ssDNA, respectively. Light blue line, chromosomal DNA; dark blue line, neosynthesized chromosomal DNA; orange dot, replisome; dark red lines, ssDNA; dark red oblong, transformation machinery; yellow circles, RecA molecules; green line, homologous DNA in chromosome. (**B**) HR pathways in competent and non-competent pneumococcal cells and implication of both RecA and RadA. In non-competent cells, HR is involved in genome maintenance pathways including replication fork restart and DNA repair. In addition, both RecA and RadA are expressed at basal levels. In competent cells, HR is still involved in replication fork restart and DNA repair, but also in transformation, where tDNA is internalized at midcell, and accesses the chromosome via active replication forks, from which homology search may emanate (6, 7). Once homology is found, HR promotes integration of tDNA into the recipient chromosome. Both *recA* and *radA* are induced during competence, resulting in increased cellular levels of RecA and RadA in competent cells. Gray half-cell, non-competent cell; green half-cell, competent cell; dark gray ovals, HR pathways; light blue line, chromosomal DNA; dark blue line, neosynthesized chromosomal DNA; orange dot, replisome; dark red line, tDNA; dark red oblong, transformation machinery; yellow circles, RecA molecules; purple circles, RadA molecules; white dots, origins of replication; black dot, terminus of replication; red dotted line, path of tDNA from internalization to integration.

Homologous recombination (HR) is central to the maintenance and plasticity of bacterial genomes. HR is a universally conserved mechanism of exchange between homologous DNA strands which is driven by the universal homologous recombinase RecA in bacteria (1). HR plays key roles in several vital pathways of genome maintenance, including replication fork restart (2) and repair of single-stranded DNA (ssDNA) gaps or double-stranded DNA (dsDNA) breaks (3). In addition, HR is essential for the widespread bacterial horizontal gene transfer mechanism of natural transformation (4). In all of these processes, HR begins with the loading of RecA onto nascent ssDNA by dedicated recombination mediator proteins (5), which promote ATP-dependent nucleofilamentation of RecA to generate a dynamic polymer known as the presynaptic HR filament. This intermediate then undergoes a homology search within the chromosome and, once a complementary sequence is found, directs ssDNA exchange to form a displacement loop (D-loop). The D-loop is then processed and resolved to restore the DNA duplex, leading to replication fork restart, repair of damaged DNA, or integration of new genetic content by transformation (Fig. 1A).

Natural transformation involves the capture and internalization of exogenous DNA into the cell cytoplasm in the form of single strands, which are then integrated into the recipient chromosome by HR (4). This promotes the acquisition of new genetic traits, notably mediating the spread of antibiotic resistance and vaccine escape. In the human pathogen *Streptococcus pneumoniae* (the pneumococcus), transformation occurs during a transient physiological state called competence, which is a tightly regulated genetic program that lasts ~20 min at the cellular level (8). During pneumococcal competence, around 100 genes are induced, including genes encoding the multiprotein transformation machinery (9–11). Pneumococcal competence is induced in response to environmental factors such as antibiotic or genotoxic stress (12–15), and has been proposed as an analog to the SOS system, which is absent in this species (16). In addition, pneumococcal competence modulates the survival of cells exposed to certain stresses including antibiotics and genotoxic agents (17, 18).

Pneumococcal competence induction occurs in two transcriptional waves, with early competence genes induced by the response regulator ComE upon its phosphorylation by the membrane-bound ComD kinase (8). Late competence genes are induced by an alternative sigma factor σ^X^, itself induced by phosphorylated ComE. Twenty-two of the >100 genes induced during competence are directly involved in transformation, and these are all controlled by σ^X^ (19). Although the majority of these are not expressed outside of competence, a few exceptions exist. These include the HR genes *recA* and *radA*, both of which encode proteins central to genome maintenance and transformation (20, 21). These genes are expressed at a basal level as well as being induced during competence (9–11). Pneumococcal cells lacking *recA* showed increased sensitivity to multiple types of DNA damages such as UV exposure or the DNA-damaging agent methyl methanesulfonate (MMS), and displayed almost no transformation activity (14, 20, 22). RadA is involved in branch migration of three-strand DNA molecules produced by RecA in *Escherichia coli* (23). Pneumococcal RadA was revealed to be a hexameric DnaB-like helicase, extending ssDNA incorporation at D-loops during transformation (21, 24). In addition, pneumococcal RadA is important for DNA damage, since cells lacking *radA* were more sensitive to MMS (21).

In non-competent pneumococcal cells, the basal expression of *recA* and *radA* provides the growing cells with a pool of RecA and RadA molecules to face replication fork restart and DNA repair events occurring on the genome (Fig. 1B). In competent cells, it is unclear if σ^X^ induction of both *recA* and *radA* expressions only serves transformation or also genome maintenance concurrently (Fig. 1B). So far, only the importance of *recA* induction for transformation has been established (25). We recently established that competence itself increases tolerance to genotoxic stress, independent of transformation (18). However, whether induction of *recA* and/or *radA* are important for this tolerance is unknown. Here, we explored the importance of the competence induction of pneumococcal *recA* and *radA* for their key roles in genome maintenance or transformation. Unexpectedly, unlike *recA*, competence induction of *radA* was not required to ensure optimal transformation. In contrast, we found that the competence induction of both *recA* and *radA* was important for competence-mediated tolerance to the genotoxic agent MMS. Competence is induced in response to cellular stresses including DNA damage (12–14), and historically has been seen as a conduit for transformation. However, recent studies have linked competence with other important cellular processes such as biofilm formation (26, 27), virulence (28–31), and transmission (32) and shown that it fosters multilevel heterogeneity within a clonal population (18). Competence is thus a general stress response of which transformation is one of multiple facets. One facet of this stress response is to increase the ability of cells to tolerate genotoxic stress, and here, we show that the competence induction of key HR mediators RecA and RadA is central to this ability.

## RESULTS

### σ^X^ *cin* box mutations prevent competence induction of the *recA* and *radA* operons

**Fig 2:**
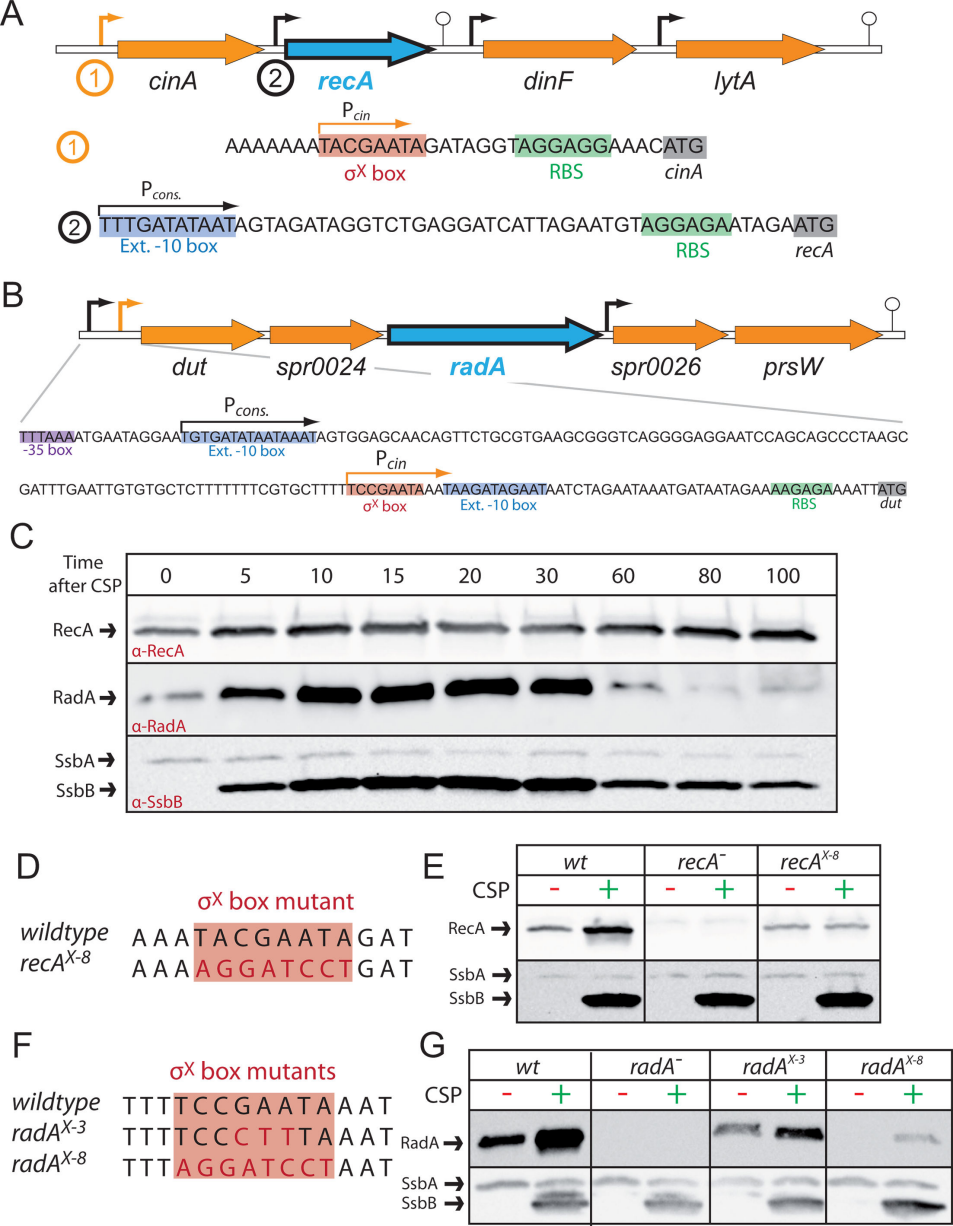
Genetic context, expression, and σ^X^ box mutants of *recA* and *radA* operons. (**A**) Genetic context of the pneumococcal *recA* locus. The *recA* gene is controlled by two promoters, a constitutive promoter (black arrow) and a competence-inducible promoter upstream of *cinA* (orange arrow). Downstream, *dinF* and *lytA* are controlled by their own promoters (black arrows), but also induced via the *cinA* competence promoter (9–11). Black arrows, constitutive promoter (P*_cons_*.); orange arrow, P*_cin_* promoter; stalked circle, terminator; gray bases, start codon; red bases, σ^X^ box; green bases, ribosome-binding site (RBS); blue bases, extended −10 box. Arrows indicate promoter orientation. (**B**) Genetic context of the pneumococcal *radA* locus. The *radA* gene is in an operon which, with *dut* and *spr0024*, is controlled by two promoters, a constitutive promoter (black arrow) and a competence promoter (orange arrow). The downstream operon consisting of *psrW* and *spr0026*, controlled by a second constitutive promoter, is also induced during competence via the same promoter. Black arrows, constitutive promoter (P*_cons_*.); orange arrow, P*_cin_* promoter; stalked circle, terminator; gray bases, start codon; red bases, σ^X^ box; green bases, RBS; purple bases, −35 box; blue bases, extended −10 box. Arrows indicate promoter orientation. (**C**) Kinetics of RecA and RadA expression before, during, and after competence. Western blot using α-RecA, α-RadA, and α-SsbB antibodies to follow protein levels in cells prior to and after exposure to CSP to induce competence. SsbB antibodies recognize both SsbB and SsbA. (**D**) Alignment of wild-type (wt) *recA* operon σ^X^ box and mutated σ^X^ box *x-8*. Red highlight, σ^X^ box; red bases, mutated bases compared to wt. (**E**) Cellular levels of RecA in competent and non-competent *x-8* σ^X^ box mutant strains. Western blot using α-RecA and α-SsbB antibodies on cellular extracts 15 min after addition (or not) of CSP to induce competence. (**F**) Alignment of wt *radA* operon σ^X^ box and mutated σ^X^ boxes *x-3* and *x-8*. Red highlight, σ^X^ box; red bases, mutated bases compared to wt. (**G**) Cellular levels of RadA in competent and non-competent *x-3* and *x-8* σ^X^ box mutant strains. Western blot using α-RadA and α-SsbB antibodies on cellular extracts 15 min after addition (or not) of CSP to induce competence.

Pneumococcal *recA* and *radA* genes are included in complex operons (33). The *recA* gene is downstream of the *cinA* gene and upstream of the *dinF* (34) and *lytA* (35) genes (Fig. 2A). Expression of *recA* is controlled by a σ^X^-induced promoter upstream of *cinA* (9–11) and a constitutive promoter upstream of *recA*. The *radA* gene is part of a three gene operon also containing the dUTP pyrophosphatase gene *dut (*11) and a gene of unknown function (*spr0024*), controlled by a constitutive promoter and a σ^X^-specific promoter (Fig. 2B). A downstream operon consisting of genes encoding the essential pneumococcal carbonic anhydrase Pca (36) and the degenerate protease PrsW (37) is also controlled by the same competence promoter. No other proteins produced by these two operons are involved in transformation, although a link between CinA and transformation has been suggested (38). Competence induction involves a peptide autoinducer called CSP (competence-stimulating peptide), encoded by the *comC* gene. The strains used in this study possess a *comC0* deletion, which prevents the production of endogenous CSP. As a result, cell populations can only become competent by addition of exogenous CSP, which artificially synchronizes a population to competence (18), limiting heterogeneity and allowing direct comparison of fully competent or non-competent populations.

The fact that both *recA* and *radA* are controlled by two promoters, one constitutive and one competence-inducible, suggests that basal expression of these genes is sufficient to ensure genome maintenance in non-competent cells, but that a boost in cellular levels is required to ensure transformation in competent cells and possibly genome maintenance during this transient differentiation state. The induction of *recA* and *radA* during competence is dependent on the alternative sigma factor σ^X^, which recognizes an 8 bp sequence (σ^X^ *cin* box) in the promoters (10, 39). Comparing the production of RecA and RadA to the competence-specific protein SsbB (40, 41) and its basally expressed paralog SsbA (42) after competence induction showed that, as expected, RecA, RadA, and SsbA were present prior to CSP addition, unlike SsbB (Fig. 2C). Once competence was induced, expression of *recA, radA,* and *ssbB* increased, reaching a peak of 20 min after CSP addition. Once competence was shut off, cellular levels of RadA and SsbB decreased as cells resumed growth, while RecA remained present at higher levels in post-competent cells. This may be because the basal level of expression of *recA* is higher than *radA*, or that RecA is more stable post-competence than RadA or SsbB, as has been reported previously for certain other competence proteins (43). To explore the importance of competence induction of HR genes *recA* and *radA* in transformation and genome maintenance, we generated strains with σ^X^ box mutations designed to abrogate the competence induction of the *recA* and *radA* genes. Replacing the eight bases of the *recA* σ^X^ box (*recA^X-8^*; Fig. 2D) resulted in loss of competence induction without altering basal expression (Fig. 2E), and this strain was compared with wild-type (wt) and *recA*^−^ strains in this study. In contrast, replacing the eight bases of the *radA* σ^X^ *cin* box (*radA^X-8^*; Fig. 2F) strongly affected competence induction but also affected basal expression (Fig. 2G). This may be due to the proximity of the basal and competence promoters of *radA*, along with the fact that the basal promoter is upstream of the competence promoter (Fig. 2B), unlike for *recA* (Fig. 2A). Replacing three central bases of the *radA* σ^X^ box (*radA^X-3^*; Fig. 2F) reduced *radA* expression to a lesser extent than *radA^X-8^*, with again minor competence induction observed (Fig. 2G). As a result, since neither strain represented a perfect abrogation of *radA* competence induction, both strains were studied in parallel with wt and *radA*^−^ strains in this study. Complementation strains with *recA* or *radA* expressed ectopically from the isopropyl β-D-1-thiogalactopyranoside (IPTG)-inducible *P*_lac_ promoter (28) at the CEP (CEP_lac_*-recA*, CEP_lac_*-radA*) expression platform (44) were generated (Fig. S1A and B) and used when appropriate to complement competence induction mutants.

### Competence induction of the *radA* operon is not required for optimal transformation

**Fig 3:**
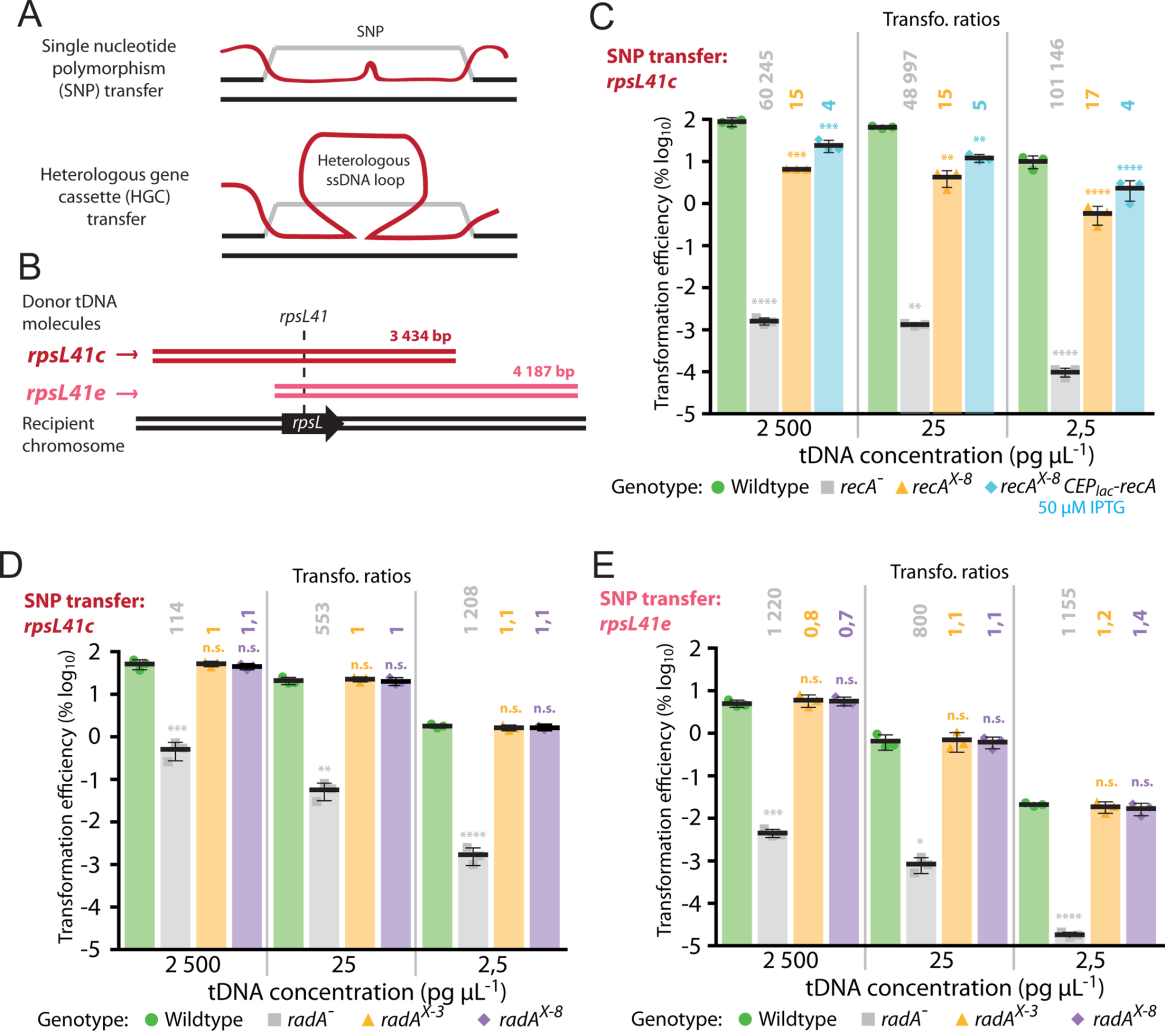
Competence induction of *recA* but not *radA* is important for transformation. (**A**) Schematic representation of chromosomal integration of SNP and HGC by transformation. These structures are resolved by replication into one wt and one transformed daughter chromosome. For HGC integration, the heterologous ssDNA loop represents the heterologous sequence, which is integrated into a daughter chromosome by replication. (**B**) Donor DNA fragments and recipient chromosome identity. Donor fragments possess the *rpsL41* point mutation, which provides streptomycin resistance (Sm^R^). In *rpsL41c* (dark red), the *rpsL41* point mutation is located centrally, while in *rpsL41e* (light red)*,* it is present at the 5´ extremity of the DNA fragment. (**C**) Competence induction of *recA* is important for transformation in both saturating (2,500 pg mL^-1^) and non-saturating (25 and 2,5 pg mL^−1^) tDNA conditions. Transformation efficiency of *rpsL41c* DNA fragments into wt (R1501, green), *recA*^−^ (R4857, gray), *recA^X-8^* (R5077, orange), and *recA^X-8^,* CEP_lac_*-recA* (R5077, blue) strains. Error bars representative of triplicate repeats, with each data point shown. Transformation ratios calculated by comparing average transformation efficiencies of test strains with wt. Statistical analyses tested significant difference between wt and test strains. ****, *P* < 0.001; ***, *P* > 0.005; **, *P* > 0.01. (**D**) Competence induction of *radA* is not necessary for optimal transformation of a point mutation central on the tDNA molecule (*rpsL41c*). tDNA in saturating (2,500 pg mL^−1^) and non-saturating (25 and 2,5 pg mL^−1^) conditions. Strains tested; wt (R1501, green), *radA*^−^ (R2091, gray), *radA^X-3^* (R4091, orange), and *radA^X-8^* (R4635, purple). Error bars representative of triplicate repeats, with each data point shown. Transformation ratios calculated and statistical analyses carried out as in panel C. Statistical analyses tested significant difference between wt and test strains. ****, *P* < 0.001; ***, *P* < 0.005; **, *P* < 0.01, n.s., not significant, *P* > 0.05. (**E**) Competence induction of *radA* is not necessary for optimal transformation of a point mutation eccentric on the tDNA molecule (*rpsL41e*). tDNA concentrations, strains, and color schemes as in panel D. Transformation ratios calculated and statistical analyses carried out as in panel C. ****, *P* < 0.001; ***, *P* < 0.005; *, *P* < 0.05, n.s., not significant, *P* > 0.05.

Two main types of transformation events exist, integration of a single nucleotide polymorphism (SNP) and integration of a heterologous gene cassette (HGC), both flanked by homologous DNA (Fig. 3A). The competence induction of *recA* was previously shown to be important for transformation using a strain where the competence promoter was inactivated by insertion of an antibiotic resistance cassette (25). To confirm this, we compared transformation of an *rpsL41* point mutation centrally located on a ~3,500 bp PCR fragment (*rpsL41c* [24]; Fig. 3B) at saturating and non-saturating conditions in wt, *recA*^−^, *recA^X-8^*, and complemented *recA^X-8^*, CEP_lac_*-recA* strains. At high tDNA concentrations (2,500 pg µL^−1^, referred to as saturating tDNA conditions hereafter), wt cells transformed at almost 100%, while only residual transformants were observed in *recA*^−^ cells (Fig. 3C), as previously reported (20). Complementation of this strain with CEP_lac_*-recA* recovered transformation efficiency to almost wt levels (Fig. S1A and C). The *recA^X-8^* strain transformed ~15-fold less than wt (Fig. 3C), showing that competence induction of *recA* was important for transformation. Complementation of this *recA^X-8^* mutation via CEP_lac_*-recA* reduced the deficit to ~4-fold compared to wt. Reducing the concentration of tDNA reduced the overall levels of transformation, confirming that the tDNA was non-saturating (25 and 2,5 pg µL^−1^), but similar differences in transformation efficiency were observed compared to wt at all concentrations tested (Fig. 3C), confirming a general importance of *recA* competence induction for transformation.

Partial complementation of *recA* inactivation by CEP_lac_*-recA* in transformation assays (Fig. S1C) may be explained by the fact that maximal expression levels of CEP_lac_*-recA* do not match those of *recA* during competence (Fig. S1A), or by a role in transformation for the competence induction of another operon member. Since CinA was previously suggested to form a complex with RecA (38), we hypothesized that competence induction of *cinA* could also be important for transformation. To explore this, we generated a *cinA* null mutant by replacing the second and third base triplets of the *cinA* sequence with stop codons (*cinA^stop^*). To confirm that this mutation resulted in loss of *cinA* expression, we tagged *cinA* and *cinA^stop^* with ALFA-tag epitope (45) and compared expression by Western blot (WB) in competent and non-competent cells. Results confirmed that the *cinA^stop^* mutation abrogated *cinA* expression (Fig. S1D). In addition, Western blot analysis confirmed that this mutant did not affect the competence induction of neighboring *recA* (Fig. S1E). This mutant was shown to transform at levels comparable to wt (Fig. S1F). This suggests that rather than an effect on *cinA* expression, the partial complementation of *recA* inactivation by CEP_lac_*-recA* in transformation assays is due to the fact that the CEP_lac_*-recA* construct expresses *recA* at levels lower than competent wt cells (Fig. S1A).

**Fig 4:**
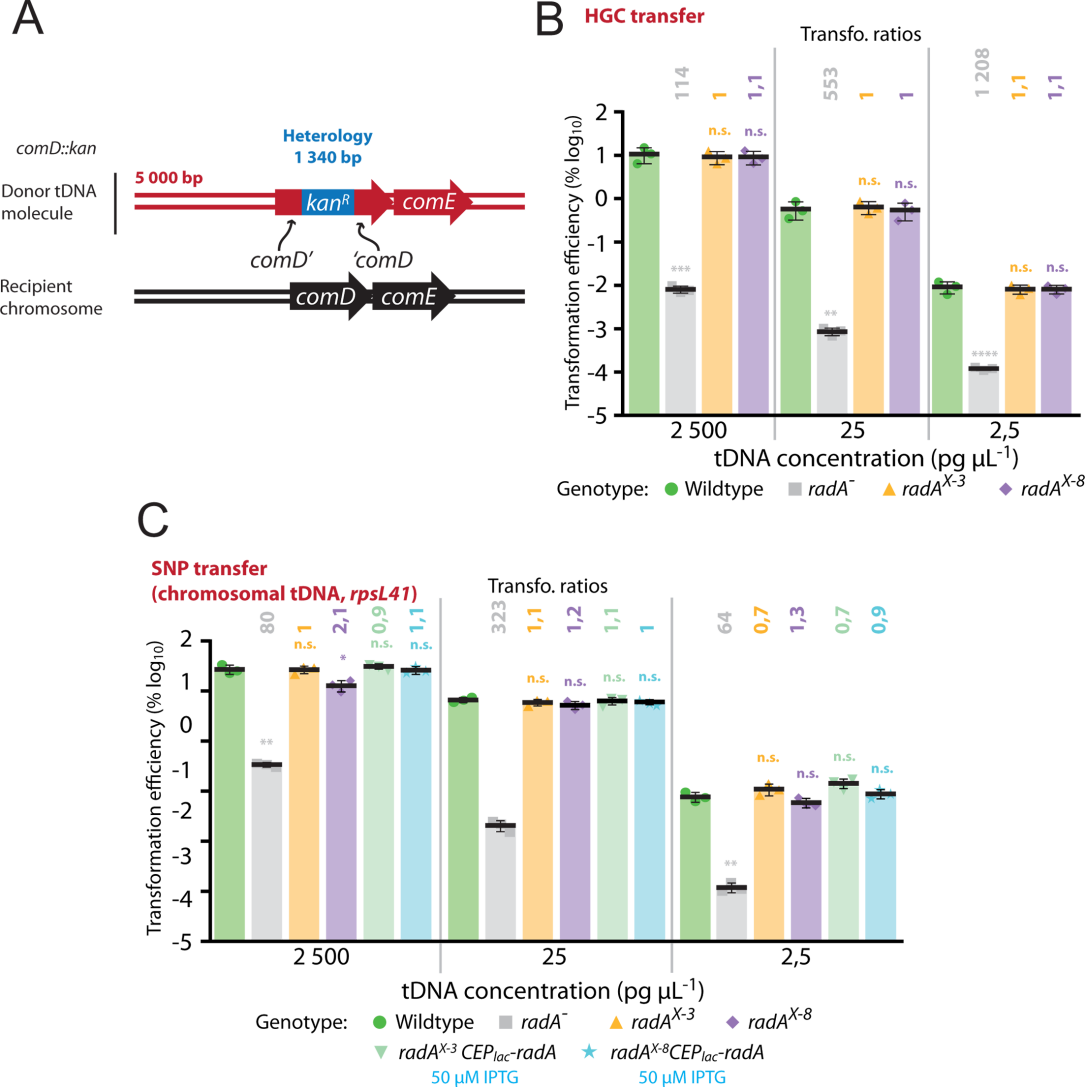
Competence induction of *radA* operon is not required for integration of heterologous cassettes by transformation. (**A**) Donor DNA fragments and recipient chromosome identity for HGC transfer. Transformation inserts a kanamycin resistance cassette into the *comD* gene, integrating 1,340 bases of heterology. (**B**) Competence induction of *radA* is not necessary for optimal transformation of heterologous cassettes. tDNA concentrations, strains, and color schemes as in Fig. 3D. Experiments carried out in triplicate with individual data points, means, and standard deviations presented. Transformation ratios calculated and statistical analyses carried out as in Fig. 3C. ****, *P* < 0.001; ***, *P* < 0.005; **, *P* < 0.01, n.s., not significant, *P* > 0.05. (**C**) Competence induction of *radA* is not required for transformation of *rpsL41* point mutation from genomic DNA. tDNA concentrations as in Fig. 3D. Strains tested; wt (R1501, green), *radA*^−^ (R2091, gray), *radA^X-3^* (R4091, orange), and *radA^X-8^* (R4635, purple), *radA^X-3^,* CEP_lac_*-radA* (R4091, light green), and *radA^X-8^,* CEP_lac_*-radA* (R4635, light blue). Experiments carried out in triplicate with individual data points, means, and standard deviations presented. Transformation ratios calculated and statistical analyses carried out as in Fig. 3C. **, *P* < 0.01; *, *P* < 0.05, n.s. (not significant), *P* > 0.05.

Since *radA* is specifically induced during competence (9–11), and key for transformation (21, 24), we hypothesized that the induction of *radA* could be similarly important for transformation. To explore this hypothesis, we repeated the transformation assay with wt, *radA*^−^, *radA^X-3^*, and *radA^X-8^* strains transformed with the ~3,500 bp PCR fragment *rpsL41c* as tDNA. When saturating DNA conditions were used (2,500 pg µL^−1^), no difference in transformation efficiency was observed for either the *radA^X-3^* or the *radA^X-8^* strains compared to wt (Fig. 3D). The control *radA*^−^ strain showed a 2-log deficit in transformation efficiency compared to wt (Fig. 3D), in line with previously reported results (24). Complementation of this mutant via CEP_lac_*-radA* fully complemented the mutation, demonstrating a lack of polar effects on local gene expression and showing that inactivation of *radA* alone affected transformation efficiency (Fig. S1B and C). Competence induction of the *radA* operon remained unnecessary for optimal transformation when non-saturating tDNA concentrations were used (25 and 2,5 pg µL^−1^, Fig. 3D). RadA was previously shown to be important for extension of transformation D-loops, revealed by an increased importance for transformation of a point mutation on the extremity of a PCR fragment (*rpsL41e*, eccentric, Fig. 3B) (24). Repeating the experiment with this tDNA donor molecule revealed that competence induction of *radA* was not required for optimal transformation of eccentric point mutations either, irrespective of the tDNA concentration used (Fig. 3E). In addition, transforming cells with saturating (2,500 pg µL^−1^) or non-saturating (25 and 2,5 pg µL^−1^) concentration of a tDNA harboring a heterologous cassette providing kanamycin resistance (Fig. 4A) showed that induction of the *radA* operon was not required for efficient HGC transformation (Fig. 4B). Transforming with PCR products targets the same chromosomal location and thus theoretically generates a maximum of two concurrent D-loops on a replicating pneumococcal chromosome. However, transformation with otherwise homologous genomic DNA possessing a selectable mutation should generate many D-loops on the chromosome within a single transforming cell, which may titrate RadA and render its induction during competence important for optimal transformation. To test this, the same strains, along with two other strains complementing the lack of competence induction by ectopic, IPTG-inducible expression of *radA* from the *P*_lac_ promoter (46) (Fig. S1B), were transformed with homologous genomic DNA containing the *rpsL41c* point mutation. Transformations were carried out in saturating (2,500 pg µL^−1^) and non-saturating (25 and 2,5 pg µL^−1^) concentrations of tDNA. No differences in transformation efficiency compared to wt were observed, except for the *radA^X-8^* strain, which showed a statistically significant twofold decrease in saturating tDNA conditions (Fig. 4C). This could suggest that in these conditions, competence induction is required for optimal transformation, but since basal *radA* expression is lower in this strain (Fig. 2G) and no difference is observed for *radA^X-3^*, it is likely that a correct basal level of *radA* expression would ensure optimal transformation in these conditions. Collectively, these results suggest that the induction of *radA*, or indeed the other four genes induced by the same competence promoter, was not required for optimal transformation efficiency, even for transformation contexts in which RadA was particularly important (24) or titrated by multiple D-loops. In conclusion, competence induction of *recA* is important for transformation, while competence induction of *radA* is not.

### σ^X^ induction of *recA* and *radA* improves tolerance to MMS-mediated genotoxic stress

**Fig 5:**
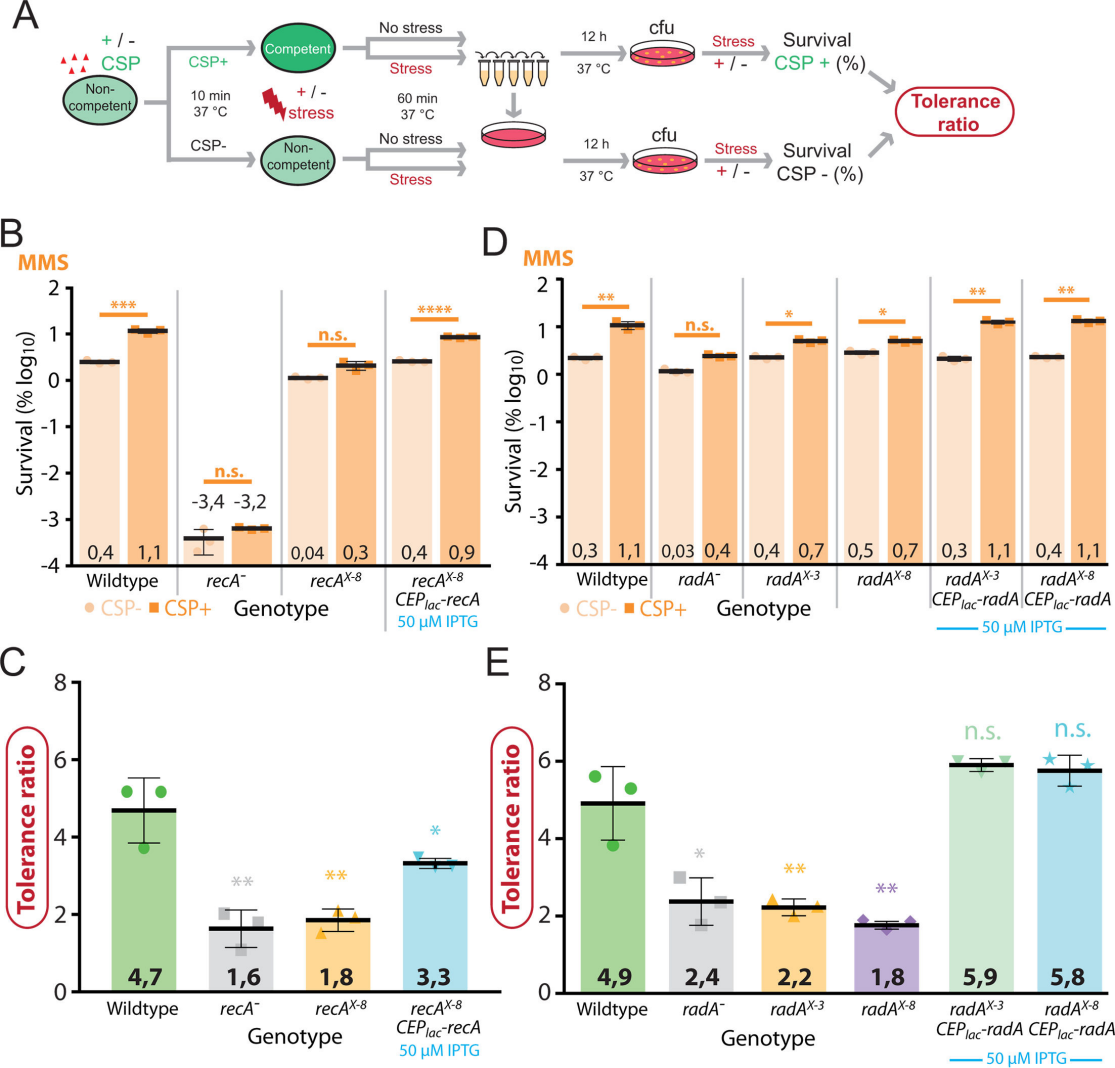
Competence induction of *recA* and *radA* is required for optimal survival of competent cells exposed to MMS. (**A**) Schematic representation of experimental procedure for survival assay used to gauge the importance of *recA* and *radA* competence induction on tolerance in response to genotoxic stresses (18). (**B**) Survival of CSP− (light orange) and CSP+ (dark orange) cells exposed to MMS (625 µg mL^-1^). Wt (R1501), *recA*^−^ (R4857), *recA^X-8^* (R5077), and *recA^X-8^,* CEP_lac_*-recA* (R5078) strains used. Experiments carried out in triplicate with individual data points, means, and standard deviations presented. Asterisks represent significance between transformation efficiencies. ***, *P* < 0.005. (**C**) Tolerance ratios of *recA* cells calculated from panel B. Asterisks represent significance between wt and mutant tolerance ratios. *, *P* < 0.05; **, *P* < 0.01. (**D**) Survival of CSP− (light orange) and CSP+ (dark orange) cells exposed to MMS (625 µg mL^−1^). Wt (R1501), *radA*^−^ (R2091), *radA^X-3^* (R4091), *radA^X-8^* (R4635), *radA^X-3^,* CEP_lac_*-recA* (R4780), *radA^X-8^,* CEP_lac_*-recA* (R4781) strains used. Representations as in panel B. Asterisks represent significance between transformation efficiencies. *, *P* < 0.05; **, *P* < 0.01. (**E**) Tolerance ratios of *radA* cells calculated from panel D. Asterisks represent significance between wt and mutant tolerance ratios. n.s., not significant, *P* > 0.05; *P* < 0.05; **, *P* < 0.01.

Next, we explored the importance of transcriptional induction of the *recA* and *radA* operons for genome maintenance by DNA repair. We recently showed that competence provides cells with increased tolerance faced with transient exposure to lethal doses of distinct genotoxic stresses (18). Although the competence protein ComM, which promotes a transient division delay in competent cells (47), played a general role in this tolerance, other unknown competence factors were also involved in increasing tolerance to such specific stresses. To determine if HR-mediated DNA repair by RecA and RadA was involved in improving tolerance of competent cells to genotoxic agents, we compared tolerance to lethal doses of MMS and norfloxacin in competent and non-competent cells in either wt cells or those lacking *recA* or *radA*, or unable to induce expression of these genes during competence. Competent or non-competent cells were exposed to these genotoxic agents at lethal concentrations (18) for 60 min, before plating to compare colony-forming units (cfu) to determine survival as a percentage. Survival of competent and non-competent populations was then compared to define a tolerance ratio, as previously defined (18) (Fig. 5A).

MMS causes dsDNA breaks and stalls replication forks by alkylating and depurinating nucleotides in DNA (48, 49), a lethal stress that we recently reported to be better tolerated by competent cells (18). Compared to wt, the *recA*^−^ strain was hyper-sensitive to MMS, and induction of competence did not increase its survival (Fig. 5B and C). Complementation via CEP_lac_*-recA* recovered survival but not to wt levels, as was observed previously for transformation (Fig. S1G and H). In light of this, the tolerance of competent and non-competent *cinA^stop^* cells was compared to explore a possible role for CinA in competence-mediated tolerance to MMS. Results showed that *cinA^stop^* cells displayed a profile of tolerance identical to wt cells, ruling out a role for CinA in this process (Fig. S1I and J). This showed that RecA was key for tolerance to MMS in both competent and non-competent cells. Also, while non-competent *recA^X-8^* cells showed similar survival levels to wt cell treated with MMS, they no longer exhibited improved tolerance to MMS upon competence induction, displaying a statistically significant difference in tolerance ratio compared to wt (Fig. 5B and C). This indicates that σ^X^ induction of *recA* was required for increased tolerance to MMS. RecA complementation via the ectopic CEP_lac_*-recA* construct recovered the tolerance increase in competent cells (Fig. 5B and C), showing that *recA* alone, rather than any other gene in the same operon (Fig. 2A), was responsible for this tolerance increase. However, there remained a significant difference between tolerance ratios of wt and complemented strains (Fig. 5C), showing that addition of basal *recA* expression and CEP_lac_*-recA* expression did not reach levels of competent wt cells, in agreement with Western blot analyses (Fig. S1A). The *radA*^−^ strain was slightly more sensitive than wt to MMS, but much less so than *recA*^−^. In addition, the competence-induced tolerance increase was significantly lower than wt, pointing to an active role for RadA along with RecA in this mechanism (Fig. 5D and E). Complementation of this strain via CEP_lac_*-radA* recovered a wt level of competence-mediated tolerance increase (Fig. S1G and H). The *radA^X-3^* and *radA^X-8^* mutant strains also showed tolerance ratios significantly lower than wt (Figure DE), showing that in the absence of σ^X^ induction of the *radA* operon, competence still increased tolerance of these strains to MMS, but significantly less than wt. Competence induction of the *radA* operon is thus important for improved tolerance to MMS. To explore whether loss of induction of *radA*, rather than any other member of the *radA* operon (Fig. 2B), was responsible for this effect, complementation mutants were tested. Indeed, these two strains (*radA^X-3^*, CEP_lac_*-radA; radA^X-8^*, CEP_lac_*-radA*) showed tolerance ratios comparable to wt, with no statistical significance observed (Fig. 5D and E), confirming that σ^X^ induction of *radA* alone was important for the competence-mediated tolerance to MMS. The competence induction of key HR genes is thus important for the increased tolerance observed in competent cells transiently exposed to lethal concentrations of the genotoxic agent MMS.

**Fig 6:**
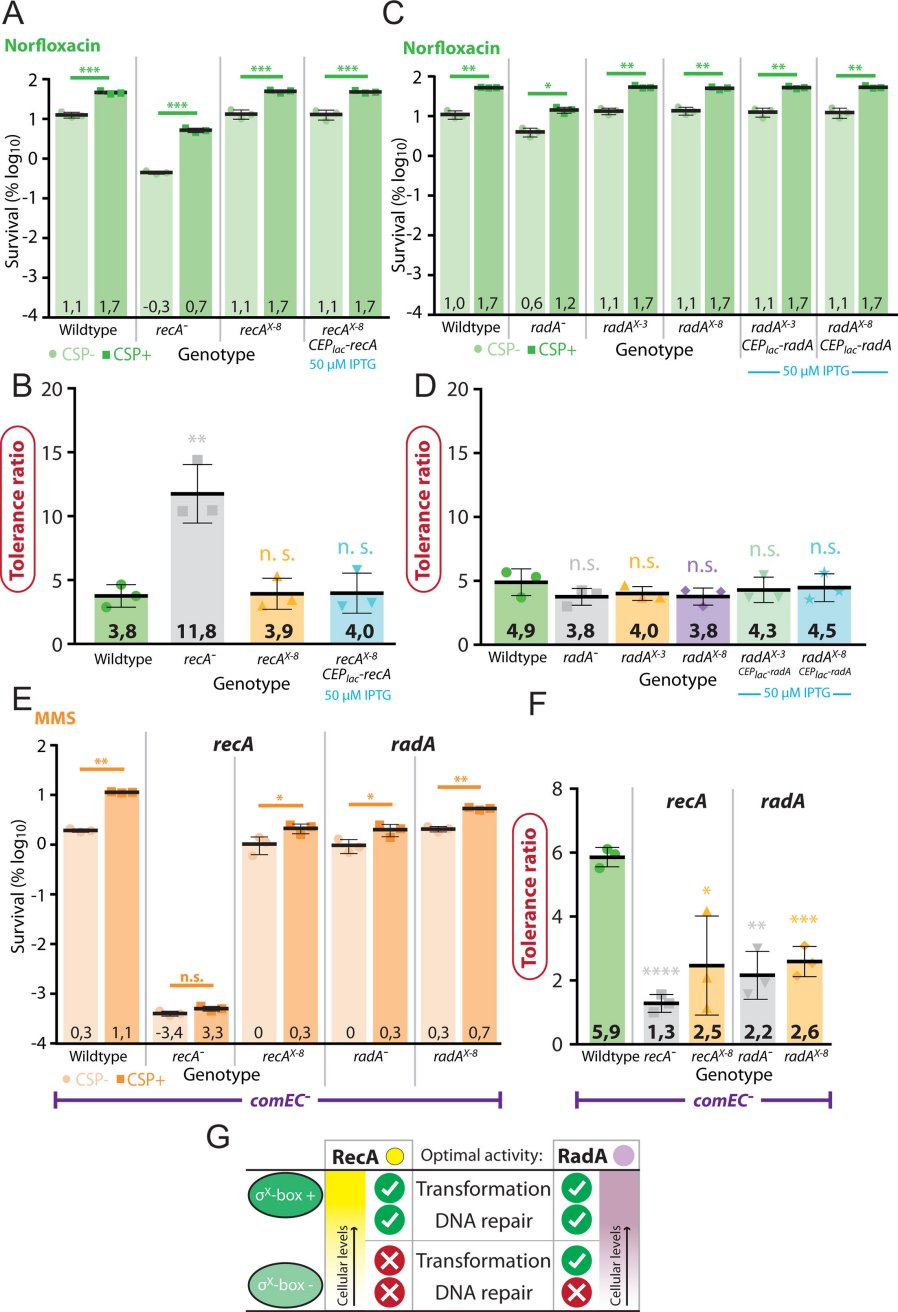
Competence induction of *recA* and *radA* is not required for optimal survival of competent cells exposed to norfloxacin. (**A**) Survival of CSP− (light green) and CSP+ (dark green) cells exposed to norfloxacin (100 µg mL^−1^). Strains and representations as in Fig. 5B. Asterisks represent significance between transformation efficiencies. n.s., not significant, *P* > 0.05; ***, *P* < 0.005; ****, *P* < 0.001. (**B**) Tolerance ratios of *recA* cells calculated from panel A. Asterisks represent significance between wt and mutant tolerance ratios. n.s.; not significant, *P* > 0.05; **, *P* < 0.01. (**C**) Survival of CSP− (light green) and CSP+ (dark green) cells exposed to norfloxacin (100 µg mL^-1^). Strains and representation as in Fig. 5D. Asterisks represent significance between transformation efficiencies. *, *P* < 0.05; **, *P* < 0.01. (**D**) Tolerance ratios of *radA* cells calculated from panel C. Asterisks represent significance between wt and mutant tolerance ratios. n.s.; not significant. (**E**) tDNA acquired from lysed cells is not required for the observed competence-mediated increase in tolerance to MMS. Tolerance to MMS tested in cells lacking the *comEC* gene, encoding the transformation pore. *comEC*^−^ (R2300), *comEC*^−^ *recA*^−^ (R5187), *comEC^−^ recA^X-8^* (R5188), *comEC^−^ radA*^−^ (R4718), and *comEC^−^ radA^X-3^* (R4720) strains used. Representations as in Fig. 5B. Asterisks represent significance between transformation efficiencies. n.s., not significant, *P* > 0.05; *, *P* < 0.05; **, *P* < 0.01. (**F**) Tolerance ratios calculated from panel E. Asterisks represent significance between wt and mutant tolerance ratios. *, *P* < 0.05; **, *P* < 0.01; ***, *P* < 0.005; ****, *P* < 0.001. (**G**) Diagram representing the importance of competence induction of *recA* and *radA* on optimal transformation and DNA repair mechanisms. While competence induction of *recA* is required for both optimal transformation and DNA repair, competence induction of *radA* is required only for optimal DNA repair. Green oblongs represent competent cells with either the *recA* or σ^X^-box intact (σ^X^-box +) or mutated (σ^X^-box −).

If competence induction of *recA* or *radA* (and associated increased HR potential) was solely sufficient to promote competence-mediated MMS tolerance, strains where *cin box* inactivation is complemented via CEP_lac_ constructs should show “competent” levels of MMS tolerance even in non-competent cells, since *recA* or *radA* were constitutively produced from their native promoters as well as CEP_lac_, and thus no longer dependent on competence for expression boost. This should result in a tolerance ratio of ~1. However, for both *recA* and *radA*, strains demonstrated near wt levels of MMS tolerance for both competent and non-competent conditions, maintaining a difference between these conditions and thus a tolerance ratio of >1 (Fig. 5B through E). These observations suggested that another competence-induced factor plays a role in competence-mediated MMS tolerance in these conditions. We previously showed that the division delay-mediated competence-specific protein ComM (47) was also important for competence-mediated tolerance to MMS, as well as other stresses (18). We hypothesized that this division delay in competent cells could increase the proportion of cells able to repair sufficient DNA damage via HR to survive, potentially explaining why both competence induction of HR mediators and *comM* were required. To test this hypothesis, we inactivated *comM* (and the hydrolase *cbpD*, to which *comM* provides immunity [50, 51]) in *recA^X-8^*, CEP_lac_*-recA* cells, and repeated the MMS tolerance assay in conditions where CEP_lac_-recA expression was induced with 50 µM IPTG or not. Results show that in cells lacking *comM*, the complementation of *recA^X-8^* via CEP_lac_*-recA* no longer displays increased tolerance in competent cells, resulting in a tolerance ratio of ~1 in both inducing (IPTG+) and non-inducing (IPTG−) conditions (Fig. S2). This result strongly suggests that while an increase in HR proteins is required to increase tolerance to MMS, the transient division delay provided to competent cells by ComM is key to ensuring the beneficial effect of this HR boost. To explore whether a similar effect of competence-mediated tolerance was observed for other DNA-damaging agents, we repeated these tests with norfloxacin. Norfloxacin is an antibiotic which inhibits DNA gyrase and thus prevents DNA replication (52, 53). As shown previously (18), competent cells survived better than non-competent cells transiently exposed to the antibiotic norfloxacin at lethal concentrations (Fig. 6A and B). First, the *recA*^−^ strain showed reduced survival compared to the wt strain in both competent and non-competent conditions. However, survival remained high above that of the same strain exposed to MMS (Fig. 6A). Of note, a marked increase in survival of competent cells was observed in both wt and *recA*^−^ strains, another contrast with results observed with MMS. Thus, RecA is important for survival of cells exposed to norfloxacin, due to its involvement in HR-mediated DNA repair, but other RecA-independent pathways also exist to counteract the damaging effect of norfloxacin. In addition, the presence of RecA was not required for the competence-mediated increase in tolerance, which was significantly higher than wt (Fig. 6B), suggesting that increased protection is afforded by competence in these cells. Next, we observed that the *recA^X-8^* strain and the complemented *recA^X-8^*, CEP_lac_*-recA* strain both displayed norfloxacin survival profiles and tolerance ratios statistically indistinguishable from wt (Fig. 6A and B). This result showed that competence induction of *recA* was not required for improved tolerance to norfloxacin in these conditions. Second, a much smaller decrease in survival following norfloxacin exposure was observed for the *radA*^−^ strain in comparison to the *recA*^−^ strain (Fig. 6C), showing that RadA plays a minor role in the repair of norfloxacin-mediated damage. Both *radA^X-3^* and *radA^X-8^* mutants, as well as two complementation strains (*radA^X-3^*, CEP_lac_*-radA; radA^X-8^*, CEP_lac_*-radA*) displayed a tolerance profile and ratio statistically indistinguishable from wt (Fig. 6C and D), showing that like *recA*, the induction of *radA* during competence was not required for improved tolerance to norfloxacin in these conditions.

Taken together, these results show that the σ^X^-mediated transcriptional induction of the two key HR genes *recA* and *radA* is important for the improved tolerance of competent cells to lethal concentrations of MMS, but not norfloxacin.

### Exogenous DNA internalized by transformation is not required for tolerance to MMS

Since exposure to lethal doses of genotoxic agents kills most cells in a population, competent survivors could use homologous DNA released from neighboring cells for HR-directed chromosome repair by transformation, and access to this DNA could therefore play a role in the increased tolerance of competent cells to genotoxic stress. Indeed, a previous study suggested that addition of tDNA could improve survival of competent cells faced with the genotoxic stress mitomycin C (17). However, tDNA can cause agglutination of competent cells, so comparing survival of DNA+ and DNA− conditions using cfu can be problematic. To circumvent this issue, we inactivated the *comEC* gene, encoding the transformation pore, rendering cells unable to take up environmental DNA, and repeated the MMS tolerance assay. This allowed us to test the hypothesis of a role for exogenous DNA in tolerance without adding exogenous DNA to the cells, overcoming potential agglutination issues. Results showed that the absence of ComEC did not significantly alter the increased tolerance of competent wt cells faced with MMS (Fig. 6E and F). Accordingly, the survival and tolerance ratios of *recA* and *radA* mutants was not affected by the absence of ComEC either. Thus, we conclude that uptake of exogenous DNA by transformation is not necessary for the increased tolerance of competent cells transiently exposed to lethal doses of MMS. This shows that the DNA repair occurring in competent cells surviving lethal MMS exposure uses endogenous chromosomal DNA as a template for HR.

## DISCUSSION

In this study, we explored the competence induction of HR genes *recA* and *radA* and its importance for their roles in natural transformation and genome maintenance (Fig. 6G). We confirmed a previous observation (25) that competence induction of *recA* was required for optimal transformation efficiency. In contrast, we demonstrated that this was not the case for *radA*, where abrogation of competence induction did not significantly alter transformation efficiency, even for specific transformation events where RadA is particularly important. However, competence induction of both *recA* and *radA* was required for full competence-induced tolerance to the genotoxic agent MMS. Competence has been historically linked to transformation, but is also associated with many other processes such as biofilm formation (26, 27), virulence (28–31), and host transmission (32). Recently, we reported that competence increased tolerance to a wide variety of lethal stresses including genotoxic damages (18). The finding that transient transcriptional induction of *recA* and *radA* during competence was important for improved tolerance to genotoxic stress further reveals competence as a key process integrated into the lifestyle of the pneumococcus.

### Competence induction of *recA* and *radA* in transformation

The *recA* and *radA* genes are unusual among competence-induced genes involved in transformation in that they are each controlled by two promoters, one ensuring expression of these genes outside of competence and another ensuring expression specifically during competence (Fig. 2A and B). This may be because of their roles in both genome maintenance, which occurs in all cells, and transformation, which is restricted to competent cells. We found here that while the competence induction of *recA* was required for optimal transformation, this was not the case for *radA*. In contrast to *recA*, we were unable to generate a perfect σ^X^ *cin* box mutant of the *radA* operon, probably due to the difference in position of the basal and competence-inducible promoters (Fig. 2). However, although the competence induction of *radA* was not completely abrogated in the *radA^X-3^* and *radA^X-8^* strains, the cellular levels of RadA produced in competent cells remained below the level produced in non-competent wt cells (Fig. 2G). This shows that even when cellular RadA levels are below those of non-competent cells, optimal transformation is observed (Fig. 3 and 4). These results, taken together, suggest that high cellular levels of RecA are required for transformation, while optimal transformation can occur with very little RadA present. This difference could be explained by the fact that RecA is required for protection of tDNA after internalization (22). Preventing the boost of cellular RecA provided by competence could increase the degradation of tDNA by endogenous nucleases and in turn decrease transformation efficiency. The primary RecA-directed reaction during HR is to polymerize on ssDNA and generate a nucleofilament able to search for homology in the recipient chromosome and promote DNA strand exchange at a complementary DNA sequence. RecA should thus polymerize on each ssDNA molecule internalized during transformation. In contrast, hexamers of RadA are thought to intervene on D-loop structures, extending them in both directions (24) after chromosomal integration has occurred. This restricts the role of RadA to HR events where homology search has been successful. Thus, the cellular levels of RadA in competent cells could be less critical to the protection and recombination of tDNA compared to RecA, possibly explaining the observed difference. The fact that ectopic expression of CEP_lac_*-recA* did not fully complement the transformation deficit of the *recA^X-8^* mutation also suggests that high levels of RecA are required to ensure optimal protection of tDNA, since expression levels from the *P*_lac_ promoter are lower than during competence (Fig. S1A). In support of this hypothesis, we ruled out a role for *cinA*, induced by the same competence-inducible promoter as *recA*, in transformation (Fig. S1E). In conclusion, the competence induction of *recA* is important for optimal transformation, but this is not the case for *radA*. This is most likely due to a dose effect, with higher levels of RecA required in competent cells due to the nature of the roles of these HR proteins.

### Competence induction of *recA* and *radA* in tolerance to genotoxic stress

The competence induction of *recA* and *radA* was required for optimal tolerance to MMS, but not norfloxacin (Fig. 5 and 6). This difference may come from the nature and/or the frequency of the damages mediated by these two drugs on the genome. Norfloxacin inhibits the DNA gyrase, which blocks DNA replication (53), while MMS alkylates or depurinates nucleotides in DNA, causing base mispairing as well as replication blocks (49). Our results suggest that survival faced with MMS requires higher cellular levels of the RecA and RadA HR effectors than in the case of norfloxacin, at least in the conditions tested here. Inactivation of *recA* rendered cells much more sensitive to MMS than norfloxacin, showing that RecA-mediated HR is more important for survival of cells exposed to MMS than norfloxacin. The partial complementation of *recA*^−^ cells with CEP_lac_*-recA* also suggested that high levels of RecA are required for optimal protection from MMS (Fig. S1G and H). In addition, inactivation of *recA* reduced the tolerance ratio of cells faced with MMS, but not norfloxacin, suggesting RecA to be more important for competence-mediated MMS tolerance (Fig. 5C and 6B). Interestingly, the *recA*^−^ cells exposed to norfloxacin showed a significantly higher tolerance ratio that wild-type, demonstrating a greater survival differential between competent and non-competent populations (Fig. 6B). This shows that in the absence of RecA-mediated HR, induction of competence becomes more important, possibly due to the ComM-mediated division delay (47).

Removing *recA* or *radA* competence induction did not have the same effect on MMS tolerance. Indeed, a *recA* competence induction mutant reduced tolerance to non-competent levels (Fig. 5B and C), while both *radA* induction mutants displayed intermediate tolerance levels (Fig. 5D and E). This suggests that similarly to transformation, higher cellular levels of RecA are required for tolerance to MMS compared to RadA. This may be again due to the distinct mode of action of these two HR effectors, with RecA polymerizing on ssDNA and RadA promoting branch migration, the former requiring more molecules than the latter. In addition, while RecA is the central actor of HR, RadA is an accessory protein functionally redundant with other HR helicase such as RecG or RuvAB (54–56), which could therefore compensate for its absence. Alternatively, it may be that the minor response to competence induction in the *radA^X-3^* and *radA^X-8^* strains (Fig. 2G) results in a slight increase in tolerance. In this case, a hypothetical “true” *radA* operon σ^X^ *cin* box mutant should show a greater decrease in tolerance ratio compared to wt. The ability to take up environmental DNA by transformation was not required for competence-mediated tolerance to MMS, as shown in assays performed in a *comEC* mutant (Fig. 6E and F). This demonstrates that homologous DNA from killed neighboring cells is not used to repair the damaged genome of competent-tolerant cells by HR. As a result, HR pathways must use endogenous DNA as a template for DNA repair during tolerance to MMS, meaning that tolerant cells are self-sufficient for repairing damaged DNA by HR. Interestingly, in cells ectopically expressing *recA* or *radA,* a difference in survival was maintained between competent and non-competent cells, with levels comparable to wt in each case (Fig. 5 and 6). This showed that boosting expression levels of *recA* or *radA* was not sufficient to improve survival, and concurrent competence induction was required. This could be because boosting one of these HR effectors without the other does not increase survival when faced with MMS. Alternatively, the ComM-mediated cell division delay promoted during competence (47), which is key for increased tolerance to numerous antibiotics and genotoxic agents (18), could be necessary to give the cells time to reap the beneficial effect of HR-mediated DNA repair. The fact that inactivation of either *recA* competence induction (Fig. 5B and C) or *comM* (18) reduces the tolerance ratio to MMS to almost non-competent levels adds weight to this hypothesis. Indeed, we showed that removing *comM* in the complemented *recA* strain abrogated survival boost provided to these cells by competence (Fig. S2). This result shows that in this case, while a boost in HR effectors augments survival of cells faced with MMS, this increase is also dependent on the transient division delay provided to competent cells by ComM, possibly allowing time for a higher proportion of cells in a population to survive via HR-mediated DNA repair. This ComM-mediated delay improves tolerance to a wide variety of antibiotics and genotoxic agents targeting different cellular processes (18), and it may be that slowing down cell division provides cells with more time to overcome issues caused by stresses, with specific mechanisms involved in each case.

### Conclusions and perspectives

This study reveals that while the competence induction of the HR gene *recA* is important for transformation, this is not the case for the HR DNA branch migration gene *radA*. Pneumococcal competence has historically been linked with transformation, but recently, competence has been shown to provide many more properties to pneumococcal cells. Competence increases tolerance to many stresses, with a ComM-mediated division delay key to this (18). In addition, in this study, we identify competence induction of *recA* and *radA* genes as important for tolerance of cells faced with transient exposure to lethal doses of MMS. These results further demonstrate that competence provides cells with benefits reaching beyond transformation. As a result, competence induction of *recA* and *radA* is not exclusively for their roles in transformation. Indeed, we demonstrate here that their induction is at least equally important for some DNA repair pathways in competent cells. In all, this study reinforces the idea that pneumococcal competence provides multiple properties to a cell, including a general stress response to overcome lethal injuries, along with the ability to transform their genome and promote adaptation.

## MATERIALS AND METHODS

### Bacterial strains, competence, and transformation

The pneumococcal strains, primers, and plasmids used in this study can be found in Table S1. Standard procedures for transformation and growth media were used (57). In this study, cells were prevented from spontaneously developing competence by deletion of the *comC* gene (*comC0*) (9), rendering cells unable to produce CSP. Unless described, pre-competent cultures were prepared by growing cells to an OD_550_ of 0.1 in C + Y medium (pH 7) before 10-fold concentration and storage at –80°C as 100 µL aliquots. Antibiotic concentrations (μg mL^–1^) used for the selection of *S. pneumoniae* transformants were chloramphenicol, 4.5; erythromycin, 0.05; kanamycin (Kan), 250; spectinomycin (Spc), 100; and streptomycin. Transformation for strain construction was carried out as previously described (57), with modifications. Briefly, 100 µL aliquots of pre-competent cells were resuspended in 900 µL fresh C + Y medium with 100 ng mL^–1^ CSP (and 50 µM IPTG where necessary), then incubated at 37°C for 10 min. tDNA was then added to a 100 µL aliquot of this culture, followed by incubation at 30°C for 20 min. Cells were then diluted and plated on 10 mL casitone-tryptone (CAT) agar with 5% horse blood before incubation at 37°C for 2 h. A second 10 mL layer of CAT agar with appropriate antibiotic was added to plates to select transformants, and plates without antibiotic were included for comparison to calculate transformation efficiency. Plates were incubated overnight at 37°C. For the generation of point mutants, after exposure to DNA, cells were rediluted in 1.4 mL C + Y medium and incubated at 37°C for 3 h 30 min, before dilution and plating without selection. Successful transformants were identified by PCR and sequencing. Strains possessing the *P*_lac_ promoter platform were grown in 50 µM IPTG from the beginning of growth, as previously described (28). GraphPad Prism was used for statistical analyses, with unpaired Student’s *t*-tests used to compare wt and mutant contexts. To generate strain R2191, strain R1502 was transformed with genomic DNA from strain Δ*radA* (21), with *radA::spc* transformants selected using spectinomycin. To generate strain R2300, R1501 was transformed with a mariner mutagenesis fragment of the *comEC* gene, generated using primer pair CJ974-CJ975, with the pR410 plasmid, as previously described (58). A *comEC::kan* transformant with a kanamycin resistance cassette inserted in the *comEC* gene in a co-transcribed orientation was isolated by selection with kanamycin. To generate strain R4091, PCR fragments of regions upstream and downstream of the *radA* operon σ^X^ box were amplified, with 3 bp of the σ^X^ box mutated using primer pairs MB180-MB183 and MB181-MB182. Splicing overlap extension (SOE) PCR with these two fragments as templates and primer pair MB183-MB182 was carried out to generate a PCR fragment with the *radA* operon σ^X^ box mutated and ~2 kb of homologous sequence on either side. This DNA fragment was transformed into strain R1818 without selection, and transformants were identified by sequencing. To generate strain R4635, PCR fragments of regions upstream and downstream of the *radA* operon σ^X^ box were amplified, with 8 bp of the σ^X^ box replaced with a *Bam*HI site (GGATCC) flanked by two other bases using primer pairs DDL122-DDL123 and DDL124-DDL125. SOE PCR with these two fragments as templates using primer par DDL122-DDL125 was carried out to generate a PCR fragment with the *radA* operon σ^X^ box mutated and ~2 kb of homologous sequence on either side. This DNA fragment was transformed into strain R1501 without selection and successful transformants were identified by amplification of the region using primer pair DDL122-DDL125 followed by restriction by *Bam*HI for 1 h at 37°C. To generate strain R4660, the *radA* gene was amplified from R1501 using primer pair oALS-CJ684 possessing a *Bsp*HI site upstream and a *Bgl*II site downstream, for ligation with *Nco*I and *Bam*HI, respectively, since *radA* possessed both of those sites in its sequence. Upstream CEP_lac_ sequence flanked by *Sal*I and *Nco*I sites was amplified from R3833 using primer pair CJ588-CJ680. These two DNA fragments were ligated into the pCEP*_R_-luc* plasmid (46), digested with *Sal*I and *Bam*HI, and the ligation product was transformed directly into R1501, with transformants selected with kanamycin. To generate strain R4661, strain R4660 was transformed with a DNA fragment possessing *radA::spc* and flanking homology, amplified from strain R2191 with primer pair CJ333-CJ338, and transformants were selected with spectinomycin. To generate strain R4664, the same strategy as for strain R4660 was employed, with the *radA* gene replaced with *recA*, amplified from R1501 with primer pair CJ681-CJ682, flanking *recA* with *Nco*I and *Bam*HI restriction sites. To generate strain R4780, strain R4091 was transformed with gDNA from strain R4660, and transformants selected with kanamycin. To generate strain R4781, strain R4635 was transformed with gDNA from strain R4660, and transformants selected with kanamycin. To generate strain R5077, the *recA^X-8^* mutation (the *recA* promoter σ^X^ box replaced with AGGTACCT, also termed *cinA^cinbox-^* [43]) was amplified from strain R4446 using primer pair DDL105-DDL108 and transformed into R1501 without selection. Successful transformants were identified by amplification of the region using primer pair DDL105-DDL108 followed by restriction by *Bam*HI for 1 h at 37°C. To generate strain R5078, strain R5077 was transformed with a PCR fragment amplified from R4664 using primer pair CJ574-CJ575 and containing CEP_lac_*-recA*, with transformants selected with kanamycin. To generate strain R5326, strain R4664 was transformed with a PCR fragment amplified from strain R4857 using primer pair CJ808-CJ829 and containing *recA::trim*, with transformants selected with trimethoprim. To generate strain R5327, *comM::cat* and *cbpD::spc* cassettes were amplified with homology by PCR of strains R3967^45^ and R1620^48^ using primer pairs OCN72-OCN154 and cbpD10-cbpD11, respectively. These PCRs were co-transformed into R4781 in the presence of 50 µM IPTG, with double transformants selected with chloramphenicol and spectinomycin together. To generate strain R5328, a *cinAstop* construct, with the bases coding for the second and third amino acids of the CinA protein replaced with two stop codons (TAGTAG), was generated. Firstly, regions upstream and downstream of the *cinA* start codon were amplified, with TAGTAG added into the overlapping region of primers, using primer pairs CJ844-CJ845 and CJ846-CJ847, respectively. SOE PCR with these two fragments as templates and primer pair CJ844-CJ847 was carried out to generate a DNA fragment with *cinAstop* and ~2 kb of homologous sequence on either side. This DNA fragment was transformed into strain R1501 without selection and transformants were identified by sequencing. To generate strain R5348, upstream and downstream DNA fragments around *cinA* were amplified from R1501 DNA using primer pairs CJ992-CJ993 and CJ994-CJ995, respectively. SOE PCR with these two fragments as templates and primer pair CJ992-CJ995 was carried out to generate a DNA fragment with the *alfa* sequence downstream of *cinA*. This DNA was transformed into R1501 without selection, and successful transformants were screened using primer pair CJ992-CJ999 to detect *alfa* tag insertion. Strain R5349 was generated in the same way as strain R5348, with the exception that the initial PCRs were carried out using R5328 DNA as template, to generate a SOE PCR containing both the *cinA^stop^* and *alfa* sequence.

### Western blot

A time-course Western blot of R1501 (*comC0*) was carried out to explore the induction of *recA* and *radA* during competence. Cells were diluted 100-fold in 10 mL C + Y medium and grown to OD 0.1. A 1 mL sample was taken at *t* = 0, and competence was then induced by addition of CSP (100 ng mL^−1^). One milliliter samples were then taken 5, 10, 15, 20, 30, 60, 80, and 100 min after competence induction, with growth also measured by OD_492_ reading. One milliliter samples were centrifuged (5 min, 5,000 × *g*) and pellets were resuspended in 40 µL of Tris-EDTA buffer (TE) 1× supplemented with 0.01% sodium deoxycholate and 0.02% sodium dodecyl sulfate (SDS). Samples were then incubated for 10 min at 37°C before addition of 40 µL 2× sample buffer with 10% β-mercaptoethanol, followed by incubation at 85°C for 10 min. Samples were then adjusted to normalize the number of cells per sample using the OD_492_ readings, and loaded onto SDS-PAGE gels (BIORAD), migrated for 30 min at 200 V, and transferred onto nitrocellulose membrane using a Transblot Turbo (BIORAD). Membranes were blocked for 1 h at room temperature in 1× Tris-buffered saline (TBS) with 0.1% Tween20 and 10% milk, before two washes in 1× TBS with 0.1% Tween20. Membranes were then separated in two at the 25 kDa mark and probed with primary antibodies (1/10,000 dilution of α-RadA or α-RecA antibodies for top membrane half, 1/10,000 dilution of α-SsbB antibodies for bottom membrane half to detect both SsbB and SsbA [41]) in 1× TBS with 0.1% Tween20 and 5% milk overnight at 4°C. After a further four washes in 1× TBS with 0.1% Tween20, membranes were probed with anti-rabbit secondary antibody (1/10,000) for 1 h and 30 min, followed by another four washes in 1× TBS with 0.1% Tween20. Membranes were activated using Clarity Max ECL (BIORAD) and visualized in a ChemiDoc Touch (BIORAD). To compare the expression profiles of the *recA^X-8^* strain with wt and *recA*^−^ strains, R1501 (*comC0*), R4857 (*comC0*, *recA*^−^), and R5077 (*comC0*, *recA^X-8^*) cells were diluted 100-fold in 3 mL C + Y medium and grown to OD 0.1. Samples were split into two 1.5 mL volumes (CSP+ and CSP−) and competence was induced in CSP+ tubes by addition of CSP (100 ng mL^−1^). After 15 min at 37°C, samples were centrifuged (5 min, 5,000 × *g*) and then treated as described above. To compare the expression profiles of *radA^X-3^* and *radA^X-8^* strains with wt and *radA*^−^ strains, R1501 (*comC0*), R2191 (*comC0, radA::spc*), R4091 (*comC0, radA^X-3^*), and R4635 (*comC0, radA^X-8^*) cells were treated in the same manner. To validate the complementation of *recA* competence induction by *P*_lac_*-recA*, R1501 (*comC0*), R4857 (*comC0, recA::trim*), R5077 (*comC0, recA^X-8^*), and R5078 (*comC0, recA^X-8^, P*_lac_*-recA*) strains were diluted 100-fold in 3 mL C + Y medium (possessing or not 50 µM IPTG) and grown to OD 0.1. Samples were split into two 1.5 mL volumes (CSP+ and CSP−) and competence was induced in CSP+ tubes by addition of CSP (100 ng mL^−1^). After 15 min at 37°C, samples were centrifuged (5 min, 5,000 *g*) and then treated as described above. To validate the complementation of *radA* competence induction by *P*_lac_*-radA*, R1501 (*comC0*), R2191 (*comC0, radA::spc*), R4780 (*comC0, radA^X-3^, P*_lac_*-radA*), and R4781 (*comC0, radA^X-8^, P*_lac_*-radA*) strains were diluted 100-fold in 3 mL C + Y medium (possessing or not 50 µM IPTG as shown in Fig. 6A) and grown to OD 0.1. Samples were split into two 1.5 mL volumes (CSP+ and CSP−) and competence was induced in CSP+ tubes by addition of CSP (100 ng mL^−1^). After 15 min at 37°C, samples were centrifuged (5 min, 5,000 × *g*) and then treated as described above. To confirm that the *cinA^stop^* mutation abrogated *cinA* expression, R5348 and R5349 cells were grown at 37°C in 4 mL C + Y medium to OD_550_ 0,1. Cultures were split into two (CSP+ and CSP−), and 100 µg mL^−1^ CSP was added to CSP+ tubes. After 15 min at 37°C, samples were centrifuged (5 min, 5,000 *g*) and then treated as described above. Primary antibodies were anti-ALFA (NanoTag Biotechnologies) and anti-SsbB. To explore the effect of the *cinA^stop^* mutant on expression of *recA*, R1501 and R5328 cells were grown at 37°C in 4 mL C + Y medium to OD_550_ 0.1. Cultures were split into two (CSP+ and CSP−), and 100 µg mL^−1^ CSP was added to CSP+ tubes. After 15 min at 37°C, samples were centrifuged (5 min, 5,000 *g*) and then treated as described above. Primary antibodies were anti-RecA and anti-SsbB.

### Transformation efficiency test

To test the efficiency of transformation in wt and mutant strains, 100 µL pre-competent cultures were resuspended in 900 µL fresh C + Y medium (pH 7.6), and 100 ng mL^−1^ CSP was added. Cells were incubated for 10 min at 37°C, before 100 µL was added to tubes containing desired tDNA. tDNA identity was either a 3,434 bp *rpsL41c* fragment (24) (Fig. 3A) possessing a centrally localized point mutation conferring streptomycin resistance (amplified from R304 with primer pair MB117-MB120), a 4,187 bp *rpsL41e* fragment (24) (Fig. 3A) possessing an eccentric point mutation conferring streptomycin resistance (amplified from R304 with primer pair MB121-MB132), or a 5,000 bp fragment (Fig. 4A) possessing a Kan^R^ heterologous cassette (1 340 bp) inserted into the *comD* gene (amplified from R1745 with primer pair CJ402-CJ409). Transforming DNA was added at varying concentrations as indicated, ranging from saturating (2,500 pg mL^−1^) to very low (2.5 pg mL^−1^) concentrations. Transforming cultures were incubated at 30°C for 20 min, before dilution and plating of appropriate dilutions for selected and non-selected cells in 10 mL CAT agar medium with 3% horse blood. Plates were incubated 2 h at 37°C, before addition of a second 10 mL layer of CAT agar medium possessing either streptomycin (for *rpsL41C* and *rpsL41e* transformants) or kanamycin (for *comD::kan* transformants). Plates were incubated overnight at 37°C and colonies present on selected and non-selected plates were compared to calculate transformation efficiencies in each condition tested.

### Survival assays

To explore the effect of *recA* and *radA* competence induction on tolerance to genotoxic agents, survival assays were carried out as described previously (18), as shown in Fig. 5A. For the *recA* experiments, strains tested were R1501 (*comC0*), R4857 (*comC0, recA::trim*), R5077 (*comC0, recA^X-8^*), and R5078 (*comC0, recA^X-8^,* CEP_lac_*-radA*). For the *radA* experiments, strains tested were R1501 (*comC0*), R2191 (*comC0, radA::spc*), R4091 (*comC0, radA^X-3^*), R4635 (*comC0, radA^X-8^*), R4780 (*comC0, hexA::ery, radA^X-3^,* CEP_lac_*-radA*), and R4781 (*comC0, radA^X-8^,* CEP_lac_*-radA*). Stresses tested at levels above the minimum inhibitory concentration (18) were norfloxacin (100 µg mL^−1^) and MMS (625 µg mL^−1^). Pre-cultures were diluted 50-fold in 3 mL C + Y medium (pH 7) and incubated at 37°C until OD_550_ 0,3. These cultures were rediluted 50-fold in 5 mL fresh C + Y medium (pH 7), and incubated at 37°C until OD_550_ 0.1. Cultures were then diluted to OD_550_ 0.004 and split into two 2.5 mL samples, with competence induced in one sample by addition of 100 ng mL^−1^ CSP. Samples were then incubated at 37°C for 15 min to allow competence development, before each being split into two 1 mL samples either exposed to the genotoxic stress or not. A further incubation of 1 h at 37°C was carried out, followed by dilution and plating of cells in 10 mL CAT agar medium with 3% horse blood. Plates were incubated at 37°C overnight, and survival percentages were determined by comparing cfu of stressed and non-stressed cells. Tolerance ratios were calculated by comparing survival of CSP+ and CSP− cells.

## SUPPLEMENTAL MATERIAL

Supplemental Information mbio.03142-24-s0001.pdf Figures S1 and S2 and Table S1.

ASM does not own the copyrights to Supplemental Material that may be linked to, or accessed through, an article. The authors have granted ASM a non-exclusive, world-wide license to publish the Supplemental Material files. Please contact the corresponding author directly for reuse.
