# Supplementary material for: Competence induction of homologous recombination genes protects pneumococcal cells from genotoxic stress
Source: mBio. 2024 Nov 29;16(1):e03142-24. doi: 10.1128/mbio.03142-24 (PMC11708029; doi:10.1128/mbio.03142-24)
Supplement: Supplemental Information — Figures S1 and S2 and Table S1. [file mbio.03142-24-s0001.pdf]

Supplementary information file for:

Competence induction of homologous recombination genes protects pneumococcal cells from genotoxic stress.

David DE LEMOS[a,b], Anne-Lise SOULET[a,b], Violette MORALES[a,b], Mathieu BERGE[a,b], Patrice POLARD[a,b#], Calum JOHNSTON[a,b#].

a. Laboratoire de Microbiologie et Génétique Moléculaires (LMGM), UMR5100, Centre de Biologie Intégrative (CBI), Centre Nationale de la Recherche Scientifique (CNRS), Toulouse, France.

b. Université Paul Sabatier (Toulouse III), Toulouse, France.

# for correspondence (calum.johnston@univ-tlse3.fr, patrice.polard@univ-tlse3.fr)

File contains:

- Figure S1
- Figure S2
- Table S1

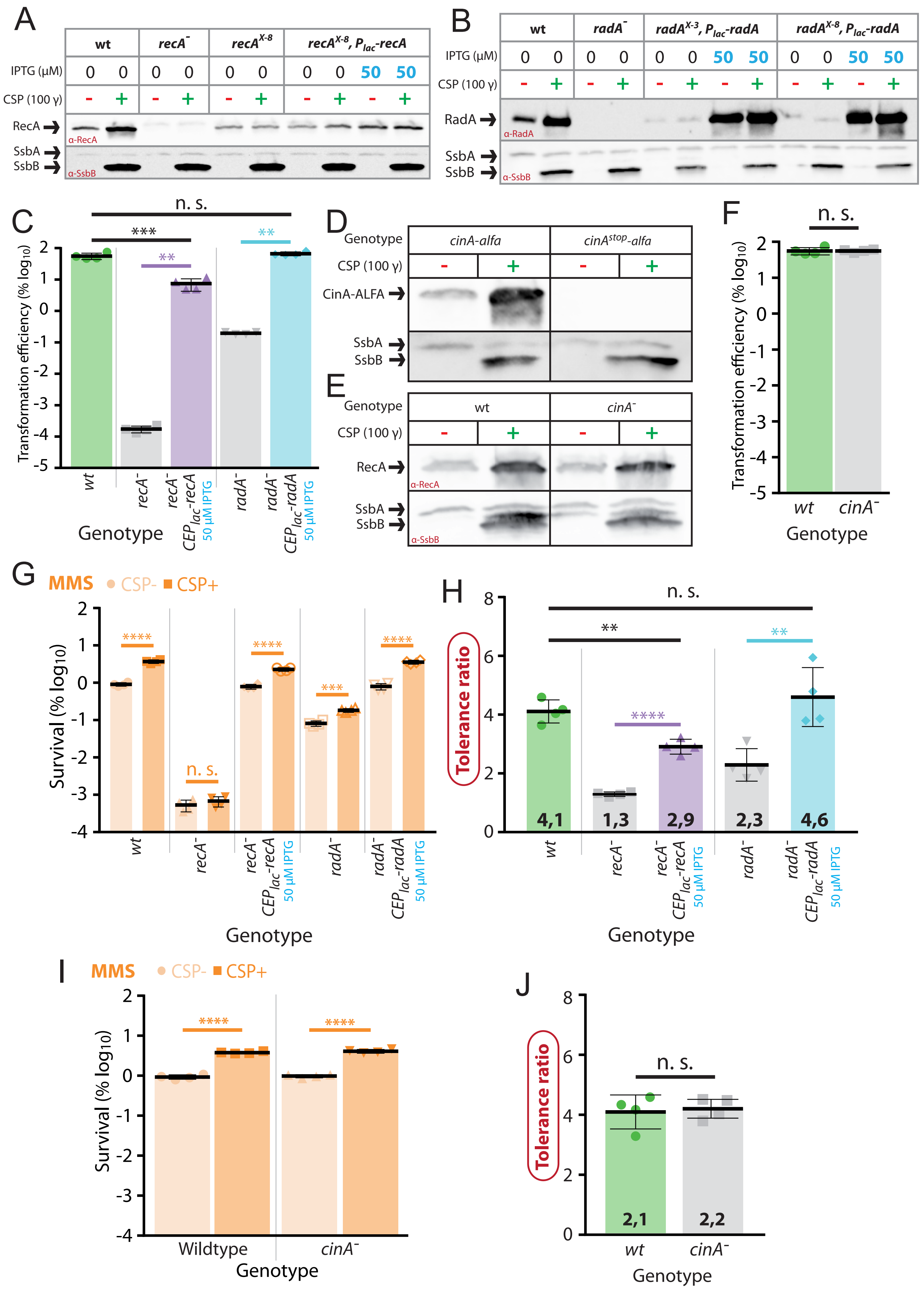

A
wt
recA-
recA X-8
recA X-8, Plac-recA
IPTG (µM)
CSP (100 γ)
RecA
SsbA
SsbB
α-RecA
α-SsbB
B
radA-
radA X-3, Plac-radA
radA X-8, Plac-radA
RadA
α-RadA
C
n. s.
Transformation efficiency (% log10)
Genotype
CEPlac-recA
CEPlac-radA
50 µM IPTG
D
cinA-alfa
cinA stop-alfa
CinA-ALFA
E
cinA-
F
G
MMS
CSP-
CSP+
Survival (% log10)
H
Tolerance ratio
4,1
1,3
2,9
2,3
4,6
I
Wildtype
J
2,1
2,2

Figure S1

Figure S1: RecA, RadA and CinA in transformation and MMS tolerance.

(A) Complementation of *recA*$^{X-8}$ strain by CEP$_{lac}$-*recA*, visualised by Western blot. Wt (R1501), *recA*$^{-}$ (R4857), *recA*$^{X-8}$ (R5077), *recA*$^{X-8}$, CEP$_{lac}$-*recA* (R5078) strains used. α–RecA antibodies used. (B) Complementation of *radA*$^{X-3}$ and *radA*$^{X-8}$ strains by CEP$_{lac}$-*radA*, visualised by Western blot. Wt (R1501), *radA*$^{-}$ (R2091), *radA*$^{X-3}$, CEP$_{lac}$-*recA* (R4780), *radA*$^{X-8}$, CEP$_{lac}$-*recA* (R4781) strains used. α–RadA antibodies used. (C) Comparison of transformation in the *recA*$^{-}$ and *radA*$^{-}$ mutant strains and strains possessing the CEP$_{lac}$ platform (induced by 50 µM IPTG) to ectopically complement these mutants. Wt (R1501), *recA*$^{-}$ (R4857), *recA*$^{-}$, *CEP*$_{lac}$-*recA* (R5326), *radA*$^{-}$ (R2091) and *radA*$^{-}$, *CEP*$_{lac}$-*radA* (R4661) strains used. Asterisks represent significance between transformation efficiencies. n. s., not significant, p > 0,05; **, p < 0,01; ***, p < 0,005. (D) Production of CinA-ALFA detected by anti-ALFA antibodies in *cinA*$^{+}$ (R5348) and *cinA*^{stop} (R5349) cells, assayed by Western blot. α-SsbB antibody used as a control to detect both SsbA (constitutive) and SsbB (competence-induced). (E) Production of RecA in competent and non-competent wt (R1501) or *cinA*$^{-}$ (R5328) cells, assayed by Western blot. α-SsbB antibody used as a control to detect both SsbA (constitutive) and SsbB (competence-induced). (F) Comparison of transformation efficiency in wt and *cinA*$^{-}$ cells. Strains as in panel E. Saturating concentrations of tDNA used (2 500 ng µL^{-1}). n. s., not significant, p > 0,05. (G) Comparison of survival in the *recA*$^{-}$ and *radA*$^{-}$ mutant strains and strains possessing the CEP$_{lac}$ platform (induced by 50 µM IPTG) to ectopically complement these mutants transiently exposed to MMS. Tolerance assay as described in Figure 5A. Strains used as in panel C. Asterisks represent significance between survival in competent and non-competent cells. n. s., not significant, p > 0,05; ***, p < 0,005; ****, p < 0,001. (H) Tolerance ratios of cells calculated from panel G. Asterisks represent significance between tolerance ratios. n.s., not significant, p > 0,05; **, p < 0,01; ***, p < 0,005; ****, p < 0,001. (I) Comparison of survival of wt and *cinA*$^{-}$ strains transiently exposed to MMS. Tolerance assay as described in Figure 5A. Strains as in panel E. Asterisks represent significance between survival in competent and non-competent cells. ****, p < 0,001. (J) Tolerance ratios of cells calculated from panel I. n.s., not significant, p> 0,05.

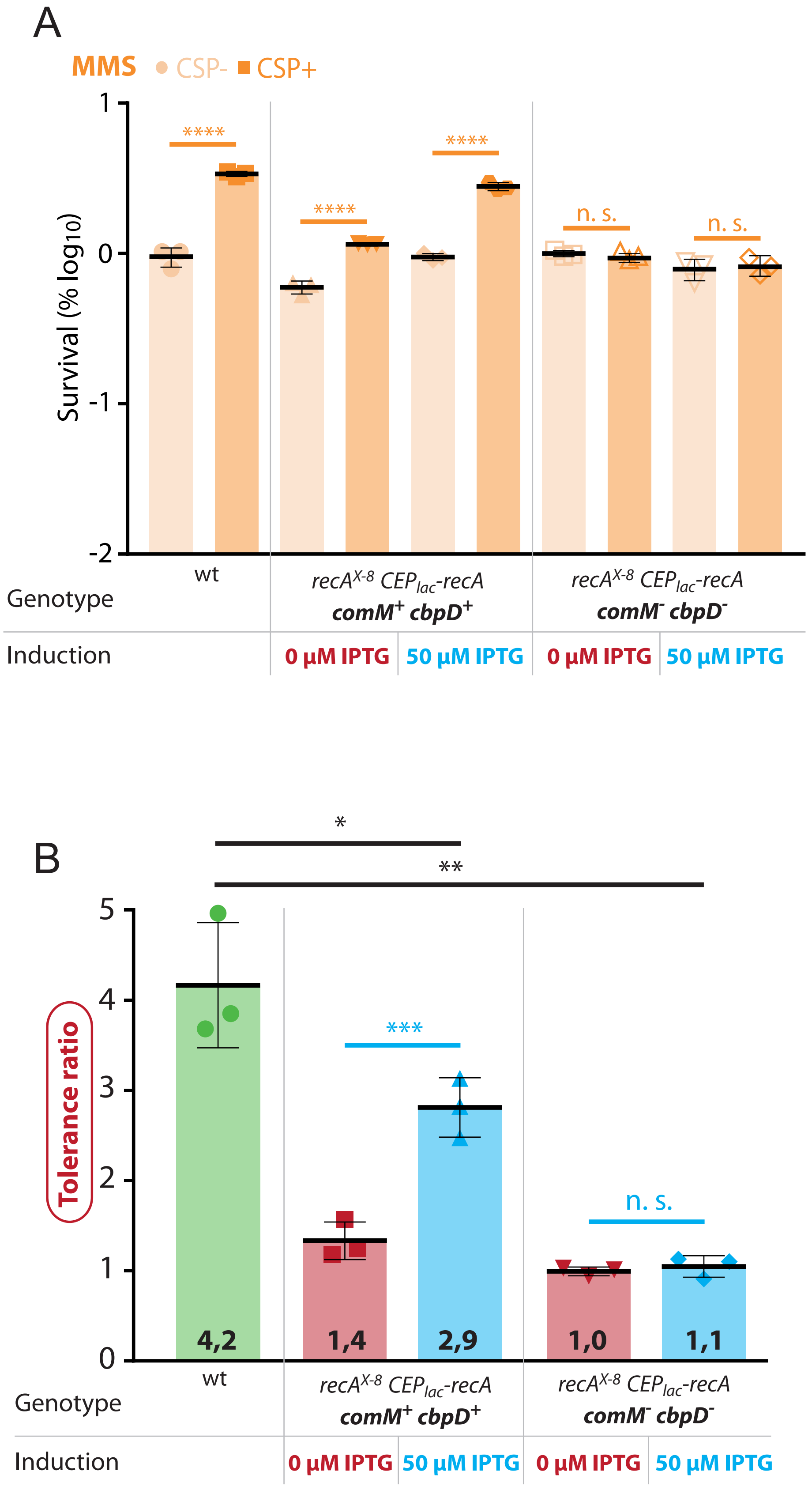
A
MMS
CSP-
CSP+
Survival (% log10)
1
0
-1
-2
****
****
****
n. s.
n. s.
wt
recA^X-8 CEP_lac-recA
comM+ cbpD+
recA^X-8 CEP_lac-recA
comM- cbpD-
Genotype
Induction
0 µM IPTG
50 µM IPTG
0 µM IPTG
50 µM IPTG
B
*
**
***
n. s.
Tolerance ratio
5
4
3
2
1
0
4,2
1,4
2,9
1,0
1,1
wt
recA^X-8 CEP_lac-recA
comM+ cbpD+
recA^X-8 CEP_lac-recA
comM- cbpD-
Genotype
Induction
0 µM IPTG
50 µM IPTG
0 µM IPTG
50 µM IPTG

Figure S2: The competence-mediated expression of both *recA* and *comM* is required for MMS tolerance.

(A) Comparison of survival of strains transiently exposed to MMS, as described in Figure 5A. Strains used, wt (R1501), *recA*$^{X\text{-}8}$, *CEP*$_{lac}$*-recA* (R5078), *recA*$^{X\text{-}8}$, *CEP*$_{lac}$*-recA*, *comM::cat*, *cbpD::spc* (R5327). Strains were either grown in 0 µM IPTG or 50 µM IPTG, as shown. Asterisks represent significance between survival in competent and non-competent cells. n.s., not significant, p> 0,05; ****, p < 0,001. (B) Tolerance ratios of cells calculated from panel A. n.s., not significant, p > 0,05; **, p < 0,01; ***, p < 0,005.

Table S1: Strains, plasmids and primers used in this study

Strain	Genotype	Source/reference
Δ radA	R6 derivative, *radA::spc* ; Spc^R	[21]
R304	*rpsL41, rif23, nov1* ; Sm^R, Rif^R, Nov^R	[25]
R800	Non-capsulated D39 derivative, 8,651 bp deletion in *cps*	[59]
R1501	*comC0*	[9]
R1502	*comC0, ssbB-luc* ; Cm^R	[9]
R1620	*rpsL1, cbpD::spc* ; Sm^R, Spc^R	[50]
R1745	*comD::kan*^{105c} ; Kan^R	[60]
R2191	*comC0, ssbB-luc, radA::spc* ; Cm^R, Spc^R	This study
R2194	*comC0, hexA::ermAM, radA::spc* ; Ery^R, Spc^R	[22]
R2300	*comC0, comEC::kan*^{2a} ; Kan^R	This study
R3967	*comC0, cbpD*^{C75A}*, comM::cat, recN::spc* ; Cm^R, Spc^R	[47]
R4091	*comC0, hexA::ery, radA*$^{X-3}$; Ery^R	This study
R4635	*comC0, radA*$^{X-8}$	This study
R4660	*comC0, CEP*$_{lac}$*-radA* ; Kan^R	This study
R4661	*comC0, CEP*$_{lac}$*-radA, radA::spc* ; Kan^R, Spc^R	This study
R4664	*comC0, CEP*$_{lac}$*-recA* ; Kan^R	This study
R4718	*comC0, hexA::ery, radA*$^{X-3}$*, comEC::kan*^{2a} ; Ery^R, Kan^R	This study
R4720	*comC0, radA*$^{X-8}$*, comEC::kan*^{2a} ; Kan^R	This study
R4780	*comC0, hexA::ery, radA*$^{X-3}$*, CEP*$_{lac}$*-radA* ; Ery^R, Kan^R	This study
R4781	*comC0, radA*$^{X-8}$*, CEP*$_{lac}$*-radA* ; Kan^R	This study
R4857	*comC0, ΔrecA::trim* ; Trim^R	[7]
R5077	*comC0, recA*$^{X-8}$	This study
R5078	*comC0, recA*$^{X-8}$*, CEP*$_{lac}$*-recA* ; Kan^R	This study
R5187	*comC0, recA*$^{-}$*, comEC::kan*^{2a} ; Kan^R	This study
R5188	*comC0, recA*$^{X-8}$*, comEC::kan*^{2a} ; Kan^R	This study
R5326	*comC0, CEP*$_{lac}$*-recA, ΔrecA::trim* ; Kan^R, Trim^R	This study
R5327	*comC0, recA*$^{X-8}$*, CEP*$_{lac}$*-recA, comM::cat, cbpD::spc* ; Kan^R, Cm^R, Spc^R	This study
R5328	*comC0, cinA*^{stop}	This study
R5348	*comC0, cinA-alfa*	This study
R5349	*comC0, cinA*^{stop}*-alfa*	This study

Plasmid	Genotype	Source/reference
pCEP$_R$-lac	pCEP derivative with the luc gene under control of P$_R$; Spc^R, Kan^R	[28]
pR412	pEMcat derivative, Amp^R, Kan^R; carries a Kan^R mariner transposon	[57]

Primer	Sequence (5'-3')	Source/reference	Use
CJ333	AAGCCCATGAAACTAGCTGAGGTG	This study	Forward primer for amplifiction of *radA::spc* DNA fragment
CJ338	CTTGTTCAGCTTTTTCAATCATGCT	This study	Reverse primer for amplifiction of *radA::spc* DNA fragment
CJ402	AACCTAACGGGCTACCGATGGCAAT	This study	Forward primer for amplifiction of *comD::kan* DNA fragment
CJ407	GAAATAGGACAACGATGGTCTTCA	This study	Reverse primer for amplifiction of *comD::kan* DNA fragment
CJ574	GCGATGCAGATTGACTTCGAACAAA	This study	Forward primer for amplification of CEP platforms
CJ575	GCGTGGAATTGACTCGATAGCTTTAAC	This study	Reverse primer for amplification of CEP platforms
CJ588	GCG*GTCGAC* AAAAAAAGAAAGGACGAAAT	This study	Forward primer for amplification of *CEPlac* upstream region
CJ680	GCG*CCATGG* AATTGTTATCCGCTCACAATTCCACA	This study	Reverse primer for amplification of *CEPlac* upstream region
CJ681	GCG*CCATGG* AAGGAGGTGTACATATGTTTGTTGCGAGAGATGC	This study	Forward primer for amplification of *recA* to generate *CEP$_{lac}$-recA*
CJ682	GCG*GGATCC* CTATTCTACCATATTTTTCAAG	This study	Reverse primer for amplification of *recA* to generate *CEP$_{lac}$-recA*
CJ684	GCG*AGATCT* TTATGCAAAGACCTTTTTCAAA	This study	Reverse primer for amplification of *radA* to generate *CEP$_{lac}$-radA*
CJ808	GCGAACTCCTTTATTTCTACCTTACAAA	[7]	Reverse primer for amplification of Δ*recA::trim*
CJ829	GCGTGGAGCAGAGCTTGCTATAGAAACC	[7]	Forward primer for amplification of Δ*recA::trim*
CJ844	GCGATGGATGTTATGATGATAAGTTTAG	This study	Forward primer for amplifiction of *cinAstop* DNA fragment
CJ845	AATCTCTGTTCCAACAGCAATGATTTC**CTACTA**CATGTTTCCTCCTACCTATCTATTCGT	This study	Reverse primer for insertion of *cinAstop* mutation by SOE
CJ846	ACGAATAGATAGGTAGGAGGAAACATG**TAGTAG**GAAATCATTGCTGTTGGAACAGAGATT	This study	Forward primer for insertion of *cinAstop* mutation by SOE
CJ847	GCGACACAAA66AAGGGAACTGGTGATC	This study	Reverse primer for amplifiction of *cinAstop* DNA fragment
CJ974	TCCTCTTACGAATAATCTAAGAGA	This study	Forward primer for amplification of *comEC* region for mariner mutagenesis
CJ975	ATAAACTAAACCTCTTCTATCTGC	This study	Reverse primer for amplification of *comEC* region for mariner mutagenesis
CJ992	CAATGATGAGTTAGAGCGGGATAT	This study	Forward primer for amplification of *cinA-alfa* 5' fragment
CJ993	ATCTACATATTATATCAAAAGTTACTCCGTTAATCGACGCCGTAGCTCTTCCTCCAGTCGGCTTGGGTCACTTAATAAAGCCT	This study	Reverse primer for amplification of *cinA-alfa* 5' fragment
CJ994	AGGCTTTATTAAGTGACCCAAGCCGACTGGAGGAAGAGCTACGGCGTCGATTAACGGAGTAACTTTTGATATAATAGTAGAT	This study	Forward primer for amplification of *cinA-alfa* 3' fragment
CJ995	GAGATCCAGAACCACATTAACCAAGG	This study	Reverse primer for amplification of *cinA-alfa* 3' fragment
CJ997	AATCGACGCCGTAGCTCTTCCTC	This study	Reverse primer in *alfa* sequence to detect successful *cinA-alfa* transformants by PCR
DDL105	TTGCTCCGCACGTTCACCCAAACGCATGATTGATCCTTTA	[43]	Forward primer for amplifiction of *recA*$^{X-8}$ DNA fragment
DDL108	AGGAGATGGAAATATTCCTCAACAAGCAAGCTAGTTCGGA	[43]	Reverse primer for amplification of *recA*$^{X-8}$ DNA fragment
DDL122	TTATCTTGAATGATGCGCTTGATAATGGCAAACGTGTGCT	This study	Forward primer for amplifiction of *radA*$^{X-8}$ DNA fragment

Primer	Sequence (5'-3')	Source/reference	Use
DDL123	CTAGATTATTCTATCTTATT**A*<u>GGATCC</u>*** TAAAAGCACGAAAAAAAGAGC	This study	Reverse primer for insertion of *radA* $^{X\text{-}8}$ mutation by SOE
DDL124	GCTCTTTTTTTCGTGCTTTT**A*<u>GGATCC</u>*** TAATAAGATAGAATAATCTAG	This study	Forward primer for insertion of *radA* $^{X\text{-}8}$ mutation by SOE
DDL125	GGTCTCTGCATAGAGATAAAACTCACTATCAATATCACCT	This study	Reverse primer for amplifiction of *radA* $^{X\text{-}8}$ DNA fragment
MB117	AATCTCCGCTGTAGGTCACTTTCTT	[24]	Forward primer for amplifiction of *rpsl41c* DNA fragment
MB120	TTGGATTGGGTGTGCATTTGC	[24]	Reverse primer for amplifiction of *rpsl41c* DNA fragment
MB121	ACATGGACCTTCAGAGAAAGCCT	[24]	Forward primer for amplifiction of *rpsl41e* DNA fragment
MB130	CCGCGTAAATCAAAAGTAGAAAAATC	[24]	Reverse primer for amplifiction of *rpsl41e* DNA fragment
MB180	CTTTTTTTCGTGCTTTTTCC**CTT**TAAATAAGATAGAATAATC	This study	Reverse primer for insertion of *radA* $^{X\text{-}3}$ mutation by SOE
MB181	GATTATTCTATCTTATTTA**AAG**GGAAAAAGCACGAAAAAAAG	This study	Forward primer for insertion of *radA* $^{X\text{-}3}$ mutation by SOE
MB182	GCTGTCTCATATTGAATAGGACTATTAGGAA	This study	Reverse primer for amplifiction of *radA* $^{X\text{-}3}$ DNA fragment
MB183	AAGATATTACCGGAGTTCGCAATTT	This study	Forward primer for amplifiction of *radA* $^{X\text{-}3}$ DNA fragment
MB214	GGGCATAATGCGCGTGAGGA	This study	Forward primer for sequence check of *radA* $^{X\text{-}3}$ and *radA* $^{X\text{-}8}$ mutations
MB215	CCCCATCAATGACCCCAACT	This study	Forward primer for sequence check of *radA* $^{X\text{-}3}$ and *radA* $^{X\text{-}8}$ mutations
oALS35	GCG*<u>TCATGA</u>* AAGGAGGTGTACATATGGCAAAGAAAAAAGCGACATTTG	This study	Forward primer for amplification of *radA* to generate *CEP* $_{lac}$ -*radA*
OCN72	GAATGAATTCGAGACTCTCCCAACAAACTCTTGCAGAAGGA	This study	Forward primer to amplify *comM::cat* with homology
OCN154	CACCCTGCAACTGCTGAGTTTCA	This study	Reverse primer to amplify *comM::cat* with homology
cbpD10	TGAGTCAGCTTTCTCGTGGTGTAG	[47]	Forward primer to amplify *cbpD::spc* with homology
cbpD11	AGACTAAGTATCGTTCCCTCCGCT	[47]	Reverse primer to amplify *cbpD::spc* with homology

<u>Underlined, italic bases represent restriction sites</u>

Bold red bases represent those mutated compared to wt

Blue bases, alfa sequence

References

59. Lefevre JC, Claverys JP, Sicard AM. Donor deoxyribonucleic acid length and marker effect in pneumococcal transformation. J Bacteriol. 1979;138: 80–86.

60. Martin B, Soulet A-L, Mirouze N, Prudhomme M, Mortier-Barrière I, Granadel C, Noirot-Gros MF, Noirot P, Polard P, Claverys JP. ComE/ComE~P interplay dictates activation or extinction status of pneumococcal X-state (competence). Mol Microbiol. 2013; 87: 394–411. doi: 10.1111/mmi.12104 PMID: 23216914
